# Extracellular truncated tau causes early presynaptic dysfunction associated with Alzheimer’s disease and other tauopathies

**DOI:** 10.18632/oncotarget.17371

**Published:** 2017-04-22

**Authors:** Fulvio Florenzano, Corsetti Veronica, Gabriele Ciasca, Maria Teresa Ciotti, Anna Pittaluga, Gunedalina Olivero, Marco Feligioni, Filomena Iannuzzi, Valentina Latina, Michele Francesco Maria Sciacca, Alessandro Sinopoli, Danilo Milardi, Giuseppe Pappalardo, De Spirito Marco, Massimiliano Papi, Anna Atlante, Antonella Bobba, Antonella Borreca, Pietro Calissano, Giuseppina Amadoro

**Affiliations:** ^1^ European Brain Research Institute, Rome, Italy; ^2^ Institute of Translational Pharmacology, CNR, Rome, Italy; ^3^ Institute of Physics, Catholic University of the Sacred Heart, Largo F Vito 1, Rome, Italy; ^4^ Institute of Cellular Biology and Neuroscience, CNR, IRCSS Santa Lucia Foundation, Rome, Italy; ^5^ Department of Pharmacy, Pharmacology and Toxicology Section, University of Genoa, Genoa, Viale Cembrano, Italy; ^6^ Department of Neurorehabilitation Sciences, Casa Cura Policlinico, Milan, Italy; ^7^ Institute of Biostructures and Bioimaging, CNR, Catania, Italy; ^8^ Institute of Biomembranes and Bioenergetics, CNR, Bari, Italy; ^9^ Center of Excellence for Biomedical Research, University of Genoa, Genoa, Viale Benedetto XV, Italy

**Keywords:** extracellular tau, tau cleavage, Alzheimer’s disease, neurodegeneration, synapse(s), Gerotarget

## Abstract

The largest part of tau secreted from AD nerve terminals and released in cerebral spinal fluid (CSF) is C-terminally truncated, soluble and unaggregated supporting potential extracellular role(s) of NH_2_ -derived fragments of protein on synaptic dysfunction underlying neurodegenerative tauopathies, including Alzheimer’s disease (AD). Here we show that sub-toxic doses of extracellular-applied human NH_2_ tau 26-44 (aka NH _2_ htau) -which is the minimal active moiety of neurotoxic 20-22kDa peptide accumulating *in vivo* at AD synapses and secreted into parenchyma- acutely provokes presynaptic deficit in K^+^ -evoked glutamate release on hippocampal synaptosomes along with alteration in local Ca^2+^ dynamics. Neuritic dystrophy, microtubules breakdown, deregulation in presynaptic proteins and loss of mitochondria located at nerve endings are detected in hippocampal cultures only after prolonged exposure to NH _2_ htau. The specificity of these biological effects is supported by the lack of any significant change, either on neuronal activity or on cellular integrity, shown by administration of its reverse sequence counterpart which behaves as an inactive control, likely due to a poor conformational flexibility which makes it unable to dynamically perturb biomembrane-like environments. Our results demonstrate that one of the AD-relevant, soluble and secreted N-terminally truncated tau forms can early contribute to pathology outside of neurons causing alterations in synaptic activity at presynaptic level, independently of overt neurodegeneration.

## INTRODUCTION

Recently *in vitro* and *in vivo* data suggest a crucial role of extracellular tau in the initiation/progression of AD [1, 2, 3]. Accumulating evidence shows that the clearance of extracellular pathological tau forms, which represents the rationale for the promising results of the ongoing tau-based immunotherapy, is an actual AD therapeutic alternative to the not-encouraging Aβ-based pharmacological and immunological approaches [4, 5]. Although full length tau is found in cerebral spinal fluid (CSF) from both normal humans [6] and rodents [7, 8], a heterogeneous population of fragments of protein -including the NH_2_terminal and/or proline-rich domain- is for the most part detected in CSF from AD subjects [9, 10, 11, 12] and in conditioned media from AD patient-derived induced pluripotent stem cells (iPSC) cortical neurons [13]. Interestingly, the largest part of tau secreted from different *in vitro* neuronal models has been recently confirmed to be actually C-terminally truncated, soluble, unaggregated and released both by vital and dead neurons in a way independent of cell damage [14]. Pathological hyperphosphorylation and caspase-3 cleavage of tau (Asp421) taking place in diseased subjects promote its aggregation and also favor the *in vitro* secretion [15]. Besides, the N-terminal projection domain of human tau -which interacts with the plasma membrane [16] and mediates neurotoxicity mainly at synapses [17]- is required for its secretion to the extracellular space in *in situ* tauopathy model [18]. Exosomes-associated NH_2_derived tau fragments are also detected in CSF from AD patients and a different pattern of NH_2_-tau fragments in CSF may reflect disease-specific neurodegenerative processes [19]. Accordingly, passive immunization with different tau antibodies directed against the extreme and mid-region in N-terminal end of protein turns out to be the most beneficial in reducing pathological tau hyperphosphorylation and in improving cognition of aged (16-months-old) 3XTg AD mice [20], thus fostering the targeting of these selective epitopes as an actual opportunity for the cure of AD and other tauopathies. Relevantly, a comprehensive mass spectrometry (MS)-based screening carried out on CSF samples from patients affected from AD and other tauopathies has confirmed that the first NH_2_ 26-44 aminoacids of human tau are epitopes potentially targetable for promising immunotherapeutic interventions, being largely represented into proteomic profile of diseased secreted proteins [21]. However, the identification of the molecular nature of extracellular noxious tau species is of central importance for developing an effective tau-based immunotherapy and, in this framework, the biological effects of AD-relevant NH_2_-truncated human tau forms outside of neurons still remain to be determined. In addition, although a potential extracellular physiopathological role of full length tau has been highlighted, consistent with the recent findings that secreted tau is *per se* toxic on synaptic function [22, 23], no study has yet investigated whether one of the biologically relevant NH_2_-truncated forms of protein -which are largely detected in diseased CSF and released *in vivo* from AD nerve endings [9, 10, 11]- could affect the neurotransmission at the pre-synaptic level.

To this point, we previously reported that higher levels of the 20-22kDa NH_2_-truncated form of human tau - a neurotoxic fragment of the full length protein (htau40) mapping between 26 and 230 aminoacids of the longest human tau isoform- are largely detectable in presynaptic terminals [24, 25, 26] as well in peripheral CSFs from living patients affected by AD and not-AD human neurodegenerative diseases associated to dementias [27]. Importantly, following potassium-induced depolarization, this specific C-terminal truncated peptide is preferentially released from AD presynaptic nerve endings in comparison with age-matched healthy controls [28], thus providing a rationale for investigating its potential action outside of neurons. Therefore, given the physiopathological role of extracellular tau in the regulation of inter-neuronal signaling at synapses [29, 30, 31, 32] and the prevailing presence of N-terminal/middle region-containing truncated forms of protein ranging from 20-25kDa in AD-derived biological fluids [9, 10, 11, 13], we attempted to elucidate the biological effects induced by short- and long-term exposure of hippocampal isolated synaptosomes and mature primary neurons to subtoxic doses of extracellular human NH_2_htau 26-44 fragment, which is the minimal biologically active moiety of longer secreted 20-22kDa parental peptide [25, 33, 34].

## RESULTS

### NH_2_htau 26-44 (aka NH_2_htau) is highly flexible and mainly monomeric in solution

**Figure 1:**
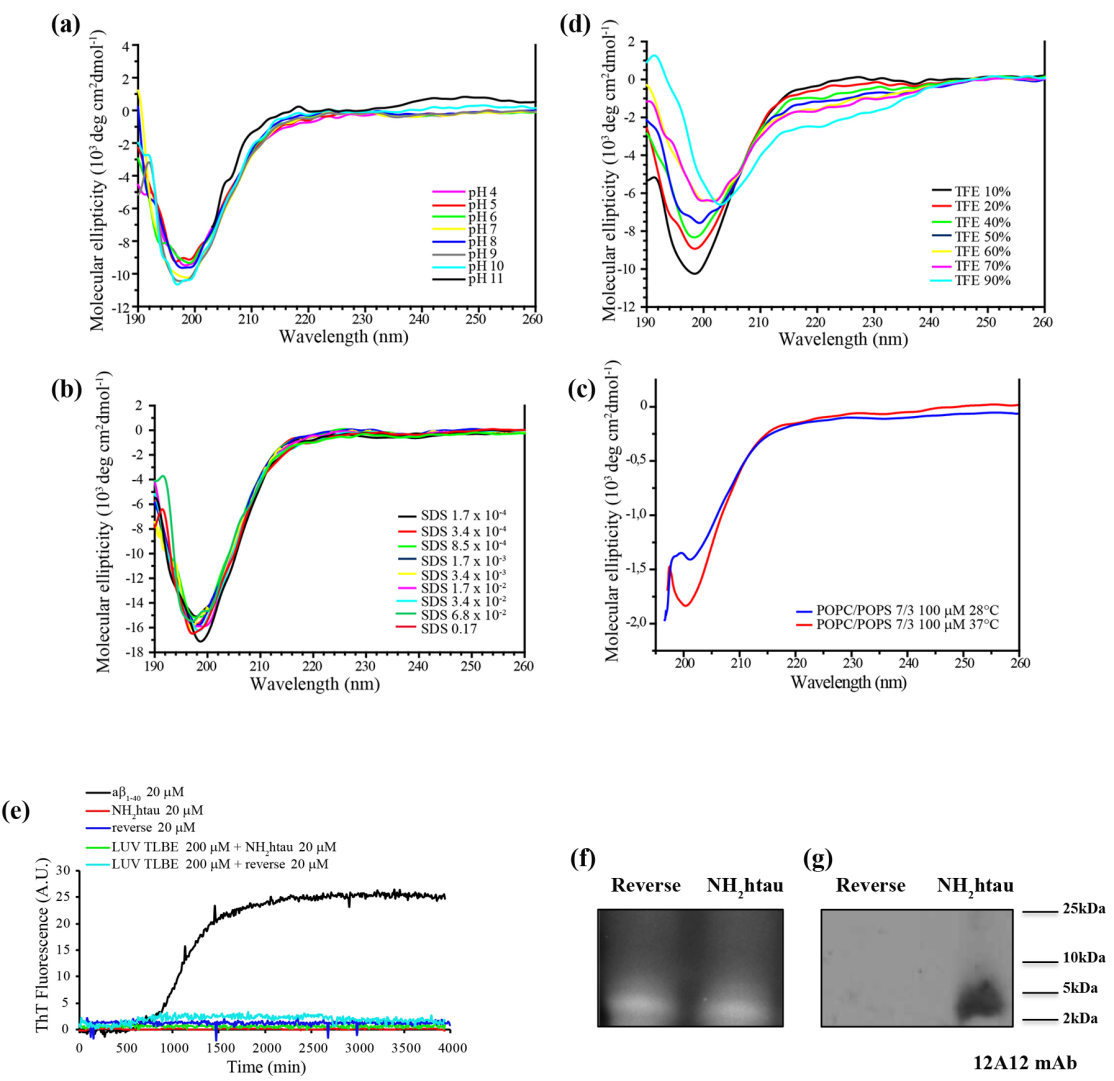
NH_2_htau 26-44 (i.e.NH_2_htau) shows an intrinsically disordered structure and is mainly monomeric in water environment **a.**-**b.**-**c.**-**d.** CD spectra of NH_2_htau26-44 (10µM ) at different pH(A), at different concentrations of SDS(B), in presence of large unilamellar vesicles (LUV) composed by POPC/POPS 100 µM in a 7/3 ratio at pH7.4 at increasing temperatures (c) and at different percentage of TFE(d) are shown. Notice that NH_2_htau shows in aqueous solution a negative peak typical for an unfolded protein with a minimum at 200nm, indicating the general absence of local major conformational changes. **e.** Kinetics of fiber formation was measured by ThT binding assay and ThT fluorescence of Aβ1-40 20 µM (black curve), NH_2_htau 20 µM in the absence (red curve) and presence of lipid LUV TLBE 200 µM (green curve), reverse control peptide (i.e reverse) 20 µM in the absence (blue curve) and presence of lipid LUV TLBE 200 µM (cyan curve) are reported. All samples were prepared in 10 mM phosphate buffer, pH 7.4, 100 mM NaCl. Time traces reported are the average of three experiments. Kinetic curves for Aβ1-40 are classically sigmoidal-shaped indicating an ongoing aggregation growth process while no noticeable differences in ThT emission intensity over time are contextually detected for NH_2_htau and reverse peptides both in solution and in presence of lipid membrane mimetics. **f.**-**g.** NH_2_htau and its reverse sequence were dissolved as described in material and methods and 150pmol of both peptides were analyzed on 15% SDS-PAGE in reducing conditions. After reversible Sypro Ruby protein staining of gel(f), Western blotting analysis was performed by probing with specific 12A12 monoclonal antibody directed against the extreme N-terminal 26-36 aa of human tau protein.Cropped representative WB is shown (g).

Although tau is an intrinsically disordered protein characterized by dynamic ensemble of interconverting conformations, recent detailed NMR spectroscopy analysis carried out on the full-length longest human tau 1-441isoform (htau40) has revealed that the first N-terminal 50 residues of protein favor a compact conformation displaying an intrinsic propensity to local secondary structure(s), as demonstrated by the presence of strong contacts within the residue stretch 1-20 and from this region to residues 30-50 [35]. In addition, the N-terminal residues Leu-Thr transiently form α-helical structure promoting a long-range intramolecular interactions [36, 37]. In view of these considerations, we set about investigating the conformational changes that chemically-synthesized (purity up to 99%) human NH_2_ 26-44 (aka NH_2_htau) and its reverse control sequence (aka reverse) undergo in aqueous solution by circular dichroism (CD) spectroscopy, which allows to estimate the secondary structural composition of proteins in different experimental environments. As shown in Figure 1a, NH_2_htau displayed within the wide range of explored pH (pH 4-11) a spectrum typical for an unfolded peptide characterized by a strong negative ellipticity below 200nm which is suggestive of a random coil unfolded conformation. No evident sign of structured peptide chains was observed in CD spectra recorded in experimental conditions similar to those occurring near the lipid bilayers (i.e.membrane-mimicking media) such as in presence of increasing detergent SDS concentrations (Figure 1b) or anionic-zwitterionic phospholipid large unilamellar vesicles (LUV) composed by POPC/POPS in a 7/3 ratio at pH7.4 (Figure 1c). Only in presence of high percentage of TFE (more than 60%), a solvent which has a dielectric constant lower than water and is widely used to mimic the hydrophobic environments of membranes, a slight propensity towards a coil-helix transition could be discernable in spectral profiles (Figure 1d). The shape and magnitude of CD curves from its reverse counterpart were not dissimilar from those of NH_2_htau (data not shown), just resembling the traditionally accepted “random coil” spectra, although conformational changes appeared more pronounced starting with lower TFE percentages (40%).

Next, in order to evaluate the capability of both tau peptides dissolved in aqueous buffer to form amyloid-like fibrils, we carried out Thioflavin T (ThT)-binding fluorescence assay. The kinetics of fiber growth in solution were monitored and time traces from NH_2_htau in the absence (red curve) and presence of LUV composed by total lipid brain extract (TLBE, green curve) were calculated. TLBE bilayers contain a physiologically important mix of lipid components -cholesterol, gangliosides, sphingolipids, isoprenoids in addition to both acidic and neutral phospholipids- making these bilayers a biologically-relevant model surface. As shown (Figure 1e), unlike the classical sigmoidal kinetic profile characterizing the ongoing Aβ1-40 fibrillogenesis which resulted in progressive 12-14 fold increase in ThT fluorescence intensity (black curve), no time-dependent emission signal was measured for NH_2_htau up to 48h. Relevantly, ThT measurements did not change over time showing the same signal recorded at time zero even after longer incubation, indicating that NH_2_htau was not prone to aggregate and was highly stable in solution. Likewise, reverse control counterpart did not show any ability to form amyloid fibrils in solution when tested both in the absence (blue curve) and presence of TLBE-LUV membrane model (cyan curve).

Taken together the above data demonstrate that both NH_2_htau and its reverse control counterpart have intrinsically unstructured disordered nature (unfolded random coil) because they neither undergo any significant structural transition in different chemical and/or membrane-mimicking environments nor form aggregation fibers over time, although CD and ThT measurements do not completely exclude the possibility that the analyzed peptides might form amorphous and/or partially structured aggregates. Consistently with its monodisperse disordered structure in aqueous solution, SDS-PAGE Western blotting analysis with *ad hoc* devised 12A12 monoclonal antibody directed against the extreme N-terminal 26-36 aa of human tau protein (Figure 1f-1g), validated that the nature of NH_2_htau was mainly monomeric with a corresponding single band that migrated more slowly than expected in a calculated mass of 2.0 kDa, in line with previous studies of disease-related disordered proteins [16, 38]. In support of the finding that the prevailing molecular form of NH_2_htau under our experimental conditions appeared to be unaggregated, we found out that the overall proportion of soluble peptide in oligomers turned out to be low as its monomeric concentration increased. A lowly-populated fraction of aggregates of higher order oligomerization migrating around 35-50kDa was faintly detected only after prolonged exposition of high-performing PVDF transfer membranes, as shown in Supplementary Figure 1.

### Extracellular-added NH_2_htau stably interacts with authentic neuronal plasma membranes under physiological conditions and perturbs artificial biomembrane-like environments in a time-dependent manner

**Figure 2:**
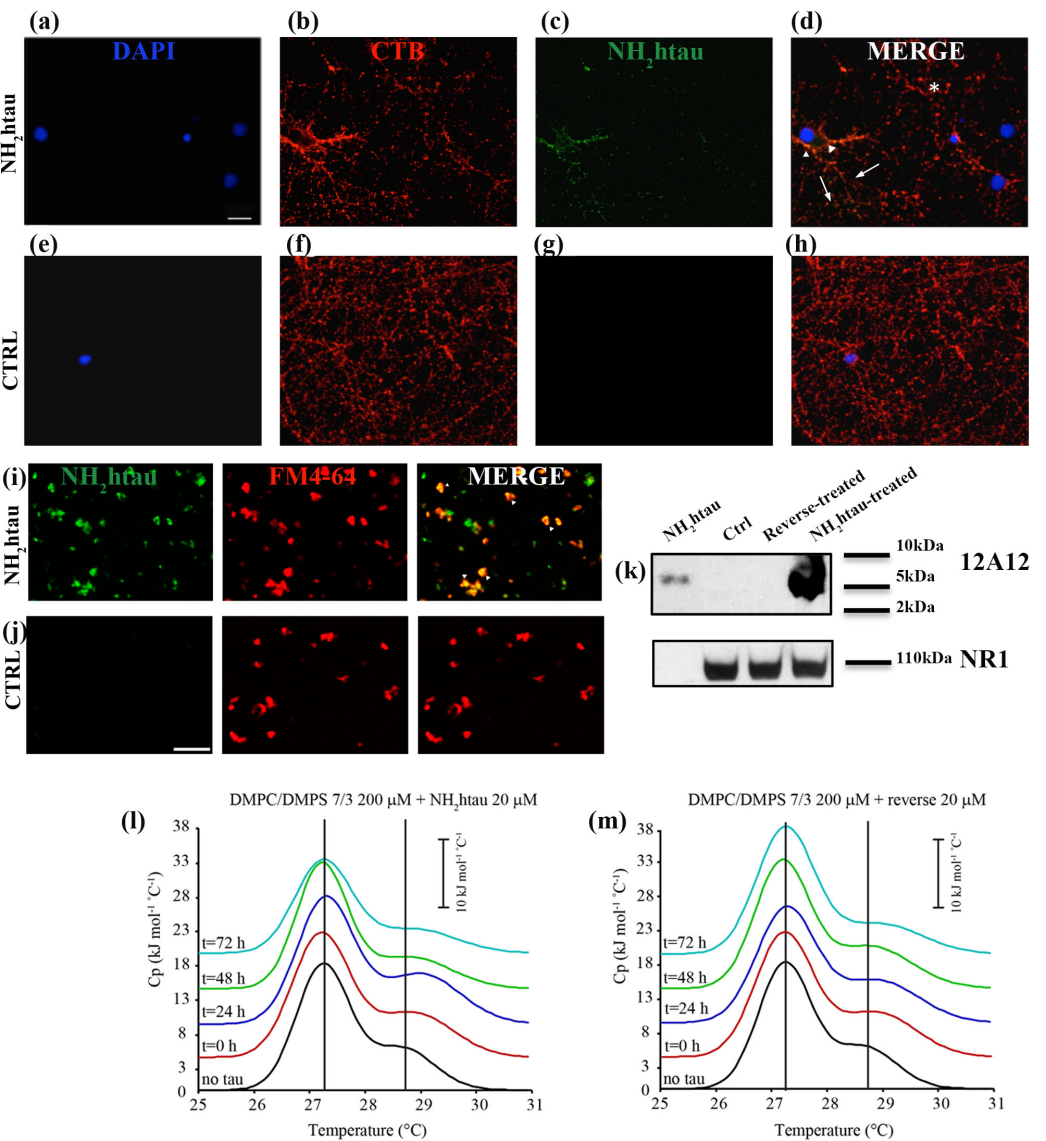
NH_2_htau is adsorbed by native neuronal plasma membranes under *in vitro* physiological conditions and deeply perturbs membrane-like lipid bilayers in a time-dependent manner **a.**-**b.**-**c.**-**d.**-**e.**-**f.**-**g.**-**h.** Fluorescence analysis (a-b-c-d) under non-permeabilizing conditions on hippocampal neurons (DIV15) in the presence or absence of the FITC-conjugated NH_2_htau. Live primary cultures were 30’exposed to FITC-conjugated NH_2_htau (1µM) (green channel) and then stained with TRITC-conjugated cholera toxin subunit b (red channel). Nuclei were stained with DAPI (blue channel). Merge image shows the composition of two fluorescence channels. Untreated cultures were used as negative control (e-f-g-h). Notice that NH_2_htau displays a diffuse, dot-like pattern distributed both in close proximity to and away from somatic compartment (arrowheads and arrows point to non-synaptic and synaptic areas, respectively). Structures which are positive for components of lipid rafts but not for NH_2_htau (asterisks) are also present. Scale bar:5 µm. **i.**-**j.** Fluorescence analysis of purified hippocampal synaptosomal fractions in the presence or absence of the FITC-conjugated NH_2_htau. Isolated synaptosomes were dual-labeled (i) by staining with FM4-64 (50µM for 1’+ 30mMKCl for 90sec) (red channel) and with FITC-conjugated NH_2_htau (1µM) for 10’(green channel). Untreated synaptosomes were used as negative control (j). Overlay image (yellow, arrowheads) indicates colocalization of NH_2_htau and FM 4-64 in numerous granular spots. Few ring-like morphological structures (usually 1–3 µm in diameter) resembling spherical synaptosomes are labeled by NH_2_htau but not by membrane-bound FM4-64 dye .Scale bar:10 µm. **k.** Western blotting analysis (n=2) of isolated membrane-containing fractions from primary hippocampal neurons (15 DIV) exposed for 1h to NH_2_htau and its reverse (5µM). Untreated cultures and synthetic NH_2_ 26-44 human tau peptide were used as controls. Immunoblotting was performed with 12A12 monoclonal antibody (26-36 aa) and with NR1 subunit antibody, to check the preparation purity. Cropped representative WB are shown. **l.**-**m.** DSC thermograms of LUV DMPC/DMPS 7/3 freshly prepared (black curve), LUV DMPC/DMPS 7/3 + NH_2_htau or reverse 20 µM each t = 0 (red curve), LUV DMPC/DMPS 7/3 + NH_2_htau or reverse 20 µM t = 24h (blue curve), LUV DMPC/DMPS 7/3 + NH_2_htau or reverse 20 µM t = 48h (green curve), LUV DMPC/DMPS 7/3 + NH_2_htau or reverse 20 µM each t = 72h (cyan curve). Values from deconvolution analysis of the peak profiles are reported in Table 1.

Despite being a highly soluble protein, full-length tau has a strong propensity to interact with lipid membranes [39, 40, 41]. Indeed, although highly charged, it selectively inserts into synthetic anionic bilayer -likely owing to the attractive electrostatic interactions between the positively charged C-terminal microtubule (MT)-binding extremity and membrane lipids- inducing structural compaction [42, 43]. Besides, the negatively charged amino-terminal projection domain of tau has been shown to physiologically interact with more detergent-resistant microdomains (lipid rafts) of the plasma membrane in a phosphorylation-dependent manner [16, 44, 45, 46]. In view of these considerations, at first we set about testing the propensity of extracellular-added NH_2_htau to associate with biological plasma membranes in a native, physiological and cellular environment by means of morphological and biochemical experimental approaches. To this aim, live imaging experiments were first carried out on mature hippocampal primary neurons (15 DIV) after exposure to FITC-conjugated NH_2_htau (1µM) for short incubation times (5’-10’) and the binding of fluorescent peptide to neuronal cell membranes was revealed under non-permeabilizing conditions by TRITC-cholera toxin subunits B which is known to specifically target the lipid raft microdomains. As shown in Figure 2a-2h, NH_2_htau-exposed cultures exhibited a disperse punctate labeling which outlined the cell periphery and consistently colocalized with membrane-bound cholera toxin, indicating that this peptide was able to bind the neuronal plasma membrane near to these detergent-resistant microdomains. This specific, dot-like pattern of immunoreactivity appeared even from shorter incubation times (5’) and was distributed both in close proximity to and away from somatic compartment (arrowheads and arrows point to non-synaptic and synaptic areas, respectively). Notably, not all structures were double-labeled for components of both lipid rafts and NH_2_htau (asterisks) and, following incubation for short period of time and under not-saturable conditions, only a few rafts-positive axons as well as synaptic boutons were decorated with fluorescent NH_2_htau. To better investigate the specific association of NH_2_htau to the synaptic terminal ends, additional colocalization studies were carried out by double confocal analysis of isolated synaptosomal preparations marked with FM4-64 -a red fluorescent dye used to image functional synaptic terminals- in the presence of FITC-conjugated NH_2_htau (1µM). As shown in Figure 2i-2j, the punctate distribution of NH_2_htau overlapped with staining of the FM4-64 -positive synaptosomes (arrowheads) supporting thus the finding that this extracellular-added peptide was able to accumulate on the plasma membrane of these subcellular compartments in exposed-neurons.

Finally, biochemical experiments of subcellular fractionation aimed to isolate the membrane-containing portions from NH_2_htau-, reverse-treated and untreated primary hippocampal cultures followed by Western blotting analysis with our 12A12 tau antibody (26-36 aa) (Figure 1f-1g), confirmed that the NH_2_htau was copurified with N-Methyl-D-aspartate (NMDA) Receptor subunit NR1-enriched fraction (Figure 2k) when extracellular-added to neurons. Control experiments were carried out to check the purity of preparation which turned out to be free of contaminations from other, non-membranous compartments (Supplementary Figure 2). Altogether, these three different experimental approaches demonstrate that the NH_2_htau fragment is able to interact with genuine neuronal membrane and synaptic boutouns only after few minutes of exposure.

Next, in order to investigate whether membrane-bound NH_2_htau is also able to perturb lipid bilayer over time, we turned to differential scanning calorimetry (DSC) which is a highly sensitive non-perturbing technique useful for a thermodynamic and kinetic characterization of lipid/protein interactions in biomimetic models [47, 48]. The potential of DSC experiments to provide useful information concerning peptide/membrane interactions is based on the fact that the heat capacity (Cp) profiles of the main transitions of mixed lipid/peptide systems can elucidate not only the effects of the peptide on the membrane but also its topological arrangement when inserted into the lipid matrix. In fact, as the enthalpy variations observed during the main lipid transitions are mainly due to the efficiency in the packing of the hydrocarbon tail, it’s possible to correlate the peptide-induced reduction of the transition enthalpy of the bilayer to the extent of the interactions between guest molecules and the core of lipid membranes. Moreover, the main transition temperature is more sensitive to interactions involving the lipid headgroups, increasing when the membrane surface is involved in the interaction with the guest peptide [49, 50, 51]. To study the dynamic interaction of both NH_2_htau and its reverse peptide with lipid bilayer, we took advantage of the same experimental setting used to analyze another intrinsically disordered protein such as α-synuclein, which is also physiologically absorbed by raft microdomains onto the neuronal membrane surface and affects the thermotropic phase behavior of anionic lipid vesicles [52, 53]. Mixed acid-zwitterionic LUV composed by DMPC/DMPS (ratio 7:3) which mimic the composition of mammalian biomembranes [54]were thus used in our studies. This artificial biomembrane model undergoes spontaneous lipid phase segregation which can be observed by DSC thermograms. In particular, calorimetric heating scans displayed two endothermic peaks characterized by separate melting temperature (Tm) of transition centred at 27.2+/-0.1 °C and 28.7+/-0.1 °C which may be ascribed to DMPC- and DMPS-rich lipid domains, respectively (Figure 2l-2m, black curves). Gaussians deconvolution of DSC thermograms was then obtained (Table 1) to calculate the enthalpy change (ΔH) values corresponding to the two melting peaks of these distinct lipid domains (21.58+/-2.15 kJ mol-1 and 8.88+/-0.9 kJ mol-1 for the DMPC- and the DMPS-rich lipid domains). The stability of the lipid bilayers over time was also tested at 25°C up to 72h and no significant change in the thermogram and thermodynamic parameters in DSC runs was detected (data not shown). Interestingly as shown in Figure 2l, the incorporation of the NH_2_htau into the DMPC/DMPS bilayers caused significant and time-dependent perturbations in their thermotropic phase behavior suggesting that this peptide was able not only to promptly associate to lipid membrane model but also to gradually induce profound structural changes in their packing. Indeed, immediately after the addition of 20 µM NH_2_htau (Figure 2l red curve) to suspension, the phase segregation was more evident and interaction of NH_2_htau with the surface of the artificial membranes was clearly distinguished by the rise in the thermal transition temperature with partitioning of two discrete domains centred at 27.2+/-0.1°C and 29.2+/-0.1 °C, respectively. Besides, we did not observe any decrease in ∆H of the two domains at t = 0 (Table 1), indicating that this extreme N-terminal tau peptide at first interacted with lipid artificial membranes on the outside, stabilizing the more rigid DMPS-based regions. Similar results were obtained after 24 hours incubation of artificial biomembrane mimetics with NH_2_htau (Figure 2l, blue curve and Table 1), as evidenced by slight changes in both enthalpy and temperature of the main transition in comparison to corresponding values at t = 0. However, a weak interaction between NH_2_htau peptide and the deep hydrophobic core of membrane appeared to be clearly discernable merely after 48 hours incubation (Figure 2l, green curve), as evidenced by diminution in both parameters of thermal transition temperature (from 29.4+/-0.1°C to 29.2+/-0.2°C) and ∆H (from 9.24+/-0.9 kJ mol-1 to 8.64+/-0.8 kJ mol-1) of DMPS-rich domains when compared to t = 24h. A more pronounced reduction in total ∆H (from 28.95+/- 2.9 kJ mol-1 to 23.49+/- 2.3 kJ mol-1) was finally detected after prolonged incubation times (72 hours, Figure 2l cyan curve) in connection with a strong drop in the ∆H of the DMPC-rich domain (from 20.31+/-2.0 kJ mol-1 to 16.94+/-1.7 kJ mol-1), as calculated by deconvolution analysis of the peaks (Table 1). Taken together these findings clearly indicate that: (i) this extreme N-terminal tau peptide enriches artificial membranes in more rigid DMPS-based regions at t = 0 and only later (t = 72h) shifts towards the more fluid, not-raft DMPC-based regions; (ii) following its immediate adsorption on the surface of lipid membrane model, the deep interaction of NH_2_htau with the hydrocarbon region takes place only after longer incubation, suggesting that this peptide is more likely to be internalized after 48-72h exposure. Differently from NH_2_htau, its reverse counterpart showed a limited effect on the thermotropic phase behavior of DMPC/DMPS membranes displaying a poor tendency to interact with model membranes and to penetrate the lipid bilayer. Indeed, in parallel set of experiments we detected that the only interaction of this control peptide was with the DMPS-rich region on the surface of the membrane -as evidenced by the increase in thermal transition temperature of the second peak (from 29.2 +/- 0.1 to 29.3+/- 0.1) - but that no concomitant decrease of the ∆H (Table 1) occurred up to 72h , suggesting the absence of any its obvious dynamic and stable interaction with the hydrophobic core of model membranes (Figure 2m). To the point, it’s noteworthy that small differences in the spatial arrangement of aminoacids within a peptide sequence have been actually proved to cause big changes in the interaction of peptide with lipid membrane by affecting in particular the peptide orientation and/or the depth of membrane insertion. Therefore considering that: (i) the guest-induced decrease of ΔH is mainly ascribable to the insertion of the externally-added molecule into the hydrocarbon core of the bilayer and (ii) the NH_2_htau incorporation into the DMPC/DMPS membrane shows an increase in the gel-liquid crystal transition temperature of the bilayer with a decreased enthalpy change, it may be concluded that- following its immediate adsorption on the surface- only the NH_2_htau has the capability to stably and deeply interact with the lipid bilayer in a time-dependent manner.

**Table 1:** Thermodinamic parameters of samples containing LUV DMPC/DMPS 7/3 ratio and NH2htau or its reverse over time.

Sample	T1 (°C)	∆H1(kJ mol^-1^)	T2 (°C)	∆H2(kJ mol^-1^)	∆Htot(kJ mol^-1^)
DMPC/DMPS 7/3 200 µM	27.2 ± 0. 1	21.58 ± 2.15	28.7 ± 0. 1	8.88 ± 0. 9	30.46 ± 3. 0
DMPC/DMPS 7/3 200 µM NH2htau 20 µM t=0 h	27.2 ± 0. 1	22.80 ± 2.3	29.2 ± 0. 1	9.68 ± 1. 0	32.48 ± 3. 2
DMPC/DMPS 7/3 200 µM NH2htau 20 µM t=24 h	27.2 ± 0. 1	23.05 ± 2.3	29.4 ± 0. 1	9.24 ± 0. 9	32.29 ± 3.2
DMPC/DMPS 7/3 200 µM NH2htau 20 µM t=48 h	27.2 ± 0. 1	20.31 ± 2.0	29.2 ± 0. 1	8.64 ± 0. 8	28.95 ± 2.9
DMPC/DMPS 7/3 200 µM NH2htau 20 µM t=72 h	27.2 ± 0. 1	16.94 ± 1.7	29.2 ± 0. 1	6.55 ± 0. 6	23.49 ± 2.3
DMPC/DMPS 7/3 200 µM reverse 20 µM t=0 h	27.2 ± 0. 1	20.78 ± 2.1	29.2 ± 0. 1	8.07 ± 0. 8	28.85 ± 2.8
DMPC/DMPS 7/3 200 µM reverse 20 µM t=24 h	27.2 ± 0. 1	21.63 ± 2.2	29.3 ± 0. 1	9.25 ± 0. 9	30.88 ± 3.1
DMPC/DMPS 7/3 200 µM reverse 20 µM t=48 h	27.2 ± 0. 1	22.14 ± 2.2	29.3 ± 0. 1	9.4 ± 0. 9	31.54 ± 3. 1
DMPC/DMPS 7/3 200 µM reverse 20 µM t=72 h	27.2 ± 0. 1	23.30 ± 2.3	29.3 ± 0. 1	8.54 ± 0. 8	31.84 ± 3.2

Collectively, these morphological and biochemical studies of live-imaging and subcellular fractionation along with biophysical DSC analyses on DMPC/DMPS lipid vesicles, demonstrate that NH_2_htau: (i) is able to interact within few minutes at least in part with lipid components located in both synaptic and non-synaptic areas of authentic neuronal plasma membranes; (ii) dynamically and spontaneously interacts with artificial membrane model, enriching earlier (up to 24h) the lipid biomimetic environments in the more rigid DMPS-based domains and being more likely internalized only after prolonged incubation times (48-72h) in correlation with its shift towards the more fluid, not-raft DMPC-based regions.

### Sublethal doses of extracellular NH_2_htau induce prompt perturbation in K^+^-evoked calcium dynamics on isolated hippocampal synatopsomes

**Figure 3:**
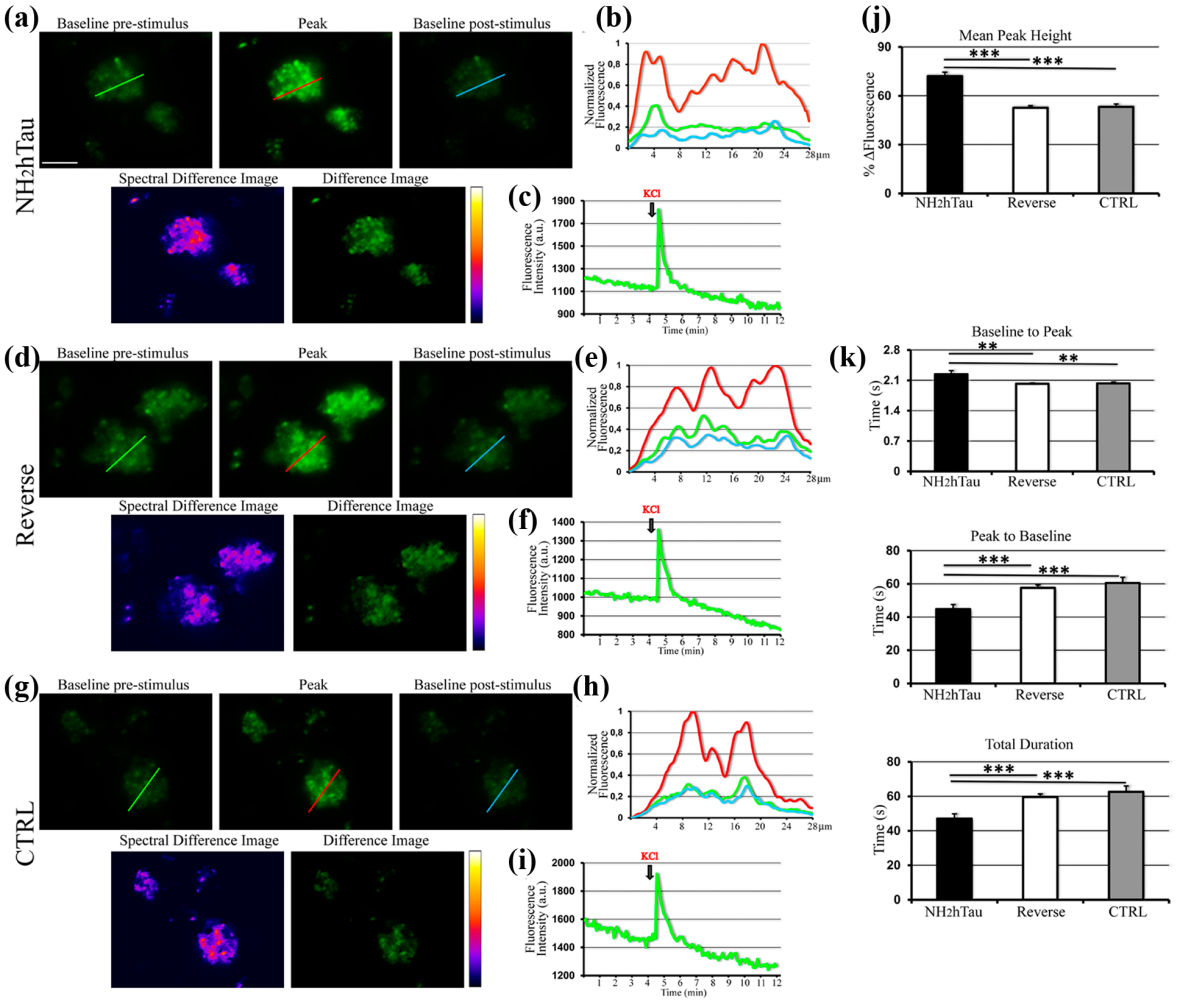
Acute administration of sub-toxic doses of NH_2_htau induces perturbation in regulation of K^+^-evoked intracellular calcium dynamics in isolated hippocampal synaptosomes Changes in K^+^-evoked Ca^2+^ levels induced by NH_2_htau and its reverse counterpart were assessed in Fluo-3 loaded hippocampal synaptosomes exposed to NH_2_htau (1µM), its reverse peptide (1µM), and saline (CTRL) 5 min before KCl (30mM) stimulation. **a.**-**d.**-**g.** Upper row: wide field images of treated synaptosomes. Baseline pre-stimulus is the image immediately before the fluorescence increase towards the peak. Peak is the image showing the highest fluorescence intensity. Baseline post-stimulus is the image immediately after that the post-stimulus baseline is established. Lower row: false color image (fire palette; Spectral Difference Image) showing the fluorescence intensity of the difference image. Brighter spots correspond to functional presynaptic terminals. Difference image is the subtraction of the baseline pre-stimulus image from the peak one. **b.**-**e.**-**h.** Normalized traces of fluorescence intensity spatial profiles derived from the line shown in a, d, and g. Green line refers to the baseline pre-stimulus image. Red line refers to the peak image. Cyan line refers to the baseline post-stimulus image. Note the relative fluorescence height intensity of the peak image (red line) which appears spatially distributed to partially fused multiple sites. **c.**-**f.**-**i.** Representative traces of fluorescence time courses derived from the three experimental groups. KCl black arrows indicate the time of the stimulus addition. **j.** Comparison, among the three experimental groups, of the average percentages of the fluorescence intensity difference between the peak and the baseline pre-stimulus values. Note the statistically significant increase of calculated value from NH_2_htau in comparison to those from the reverse and CTRL groups (one-way ANOVA followed by Bonferroni post-hoc test ***p<0,0001 versus NH_2_htau). **k.** kinetic analysis of the fluo-3 intensity time-course: baseline to peak, i.e. time for the rate of signal rise; peak to baseline, i.e. time for the rate of signal decay; total duration, i.e. the overall time from baseline pre-stimulus to baseline post-stimulus. Data were derived from five independent experiments. In each experiment two coverslips for each experimental group were analyzed. Values are means of at least four independent recordings and statistically significant differences were calculated by one-way ANOVA followed by Bonferroni post-hoc test (**p<0,01; ***p<0,0001 versus NH_2_htau). Scale bar: 20µm.

Properly controlled Ca^2+^ homeostasis is crucial for physiological maintenance of neuronal integrity as well as for survival and synaptic plasticity by controlling the release of neurotransmitter, membrane excitability and network activity. Consequently, perturbations in Ca^2+^ signaling have been proposed as one of the earliest age-related event underlying the synaptic pathology occurring in the AD and leading eventually to neurodegeneration [55, 56]. Having established that neuronal plasma membrane is a plausible site of action for extracellular N-terminally truncated tau species -which are more likely to be produced inside neurons by intracellular cleavage of full length-protein and then released into the extracellular space [13, 14]- we investigated the effects induced by acute application of low concentration of extracellular NH_2_htau and its reverse peptide on both the basal and the KCl-evoked Ca^2+^ dynamics turning to quantitative imaging with highly-specific Ca^2+^-sensitive fluorescent indicator. Besides, since changes of intracellular calcium homeostasis in nerve terminals directly trigger the discharge of synaptic vesicles and glutamate release evoked by K^+^-depolarization [57, 58, 59], we performed our assays on isolated hippocampal pinched-off nerve terminals, so-called synaptosomes, which are targeted by extracellular NH_2_htau (Figure 2) and have been proved to be particularly useful for biochemical studies of presynaptic stimulus-secretion coupling [60]. To this aim, purified synaptosomal preparations were preloaded with Fluo3 dye and then 5 min exposed to NH_2_htau or its reverse counterpart at 1 µM each, a concentration which does not cause any significant *in vitro* neuronal death up to 72h -as we previously assessed by MTT assay and Western blotting detection of active cleaved caspase-3 (Supplementary Figure 3)- and more closely mimics the *in vivo* pathological range of release-competent (i.e. soluble and microtubule unbound) amount of intracellular tau [61, 62, 63, 64]. Upon chemically-induced depolarization with 30mM KCl, each Ca^2+^ transient for three different experimental groups (saline-treated ctrl, NH_2_htau- and reverse-treated) was measured and the intensity and kinetics of the intrasynaptosomal Ca^2+^ changes produced by extracellular high [K^+^] were then visualized as a characteristic rapid rise to peak followed by a descending and apparent plateau phase, consistently with previous results [65]. Quantitative averaged data of fluorescence intensity (peak amplitude, ∆F) and of kinetic parameters (peak to baseline; baseline to peak; overall duration of signal) are shown in Figure 3. Following K^+^-stimulation (Figure 3a-3b-3c-3j), synaptic fractions acutely exposed to NH_2_htau displayed a pronounced potentiation (+35% and 36%, compared to both reverse- or untreated controls) in evoked increase of free-Ca^2+^ fluorescence intensity (∆F NH_2_htau: pixel intensity 72, 07% +/-12, 33 SD, +/- 2, 4 SEM *n* = 58, one-way repeated-measures ANOVA followed by Bonferroni post-hoc test ***p <0, 0001) in comparison to both reverse- or untreated controls. On the contrary (Figure 3d-3e-3f-3g-3h-3i-3j), application of the reverse peptide failed to produce any significant change in magnitude of K^+^-induced Ca^2+^ peak height (-1%) (∆F reverse: pixel intensity, 53, 17% +/-14, 21 SD, +/- 1, 8 SEM *n* = 66, one-way repeated-measures ANOVA followed by Bonferroni post-hoc test p >0, 05) which did not significantly differ from untreated controls (∆F ctrl: pixel intensity, 52, 61% +/-10, 68 SD, +/- 1, 3 SEM *n* = 98). Further, as shown by kinetic analyses reported in Figure 3k, all the kinetic parameters displayed a significative difference when the NH_2_htau group was compared to both reverse and untreated controls. Indeed, the rise in synaptic Ca^2+^ signal (baseline to peak: NH_2_htau 2, 2 s +/-0, 42 SD, +/-0, 08 SEM; ctrl 2, 02 s +/-0, 21 SD, +/- 0, 02 SEM; reverse 2, 03 s +/-0, 34 SD, +/-0, 03 SEM) was significantly different among the three different experimental groups (+10% NH_2_htau vs ctrl and reverse, one-way repeated-measures ANOVA followed by Bonferroni post-hoc test **p <0, 01; -0, 4% ctrl vs reverse *p* >0, 05). The fall of synaptic Ca^2+^ transient (-22 % vs ctrl; -26 % vs reverse) as well as its time course (-21 % vs ctrl; -24 % vs reverse) in nerve endings preparations exposed to NH_2_htau (NH_2_htau: peak to baseline 44, 75 s +/-16, 25 SD, +/-2, 7 SEM one-way repeated-measures ANOVA followed by Bonferroni post-hoc test ***p <0, 0001; duration 47 s +/-15, 53 SD, +/-2, 8 SEM *n* = 58 one-way repeated-measures ANOVA followed by Bonferroni post-hoc test ***p <0, 0001) were significantly reduced compared to both reverse-and untreated controls. Conversely, reverse peptide (reverse: peak to baseline 60, 54 s +/-19, 62 SD, +/-3, 4 SEM; duration 62, 57 s +/-20, 15 SD, +/-3, 4 SEM *n* = 66 ) did not provoke any significant change in kinetic parameters (peak to baseline -4% vs ctrl one-way repeated-measures ANOVA followed by Bonferroni post-hoc test p >0, 05; duration -4% vs ctrl one-way repeated-measures ANOVA followed by Bonferroni post-hoc test p >0, 05) in comparison to untreated control (ctrl: peak to baseline 57, 59 s +/-14, 89 SD, +/-1, 7 SEM; duration 59, 61 s +/-14, 21 SD, +/- 1, 7 SEM). Importantly, no significant global change in Ca^2+^ amplitude over baseline was detected in parallel assays when hippocampal synaptosomes (up to 30’; data not shown) and mature (15 DIV) primary neuronal cultures as well (up to 1h; data not shown) were exposed to extracellular NH_2_htau and reverse peptide both used at the same experimental concentration (1 µM) but in the absence of KCl stimulus (resting conditions). To this point, we did not measure any significant variation in Ca^2+^ signal throughout the recording times ruling out thus the possibility that its external application could lead to other calcium-affecting mechanisms previously described for preaggregated and unaggregated full-length tau species, including ionic pore formation and membrane leakage [66, 67], activation of specific cell surface receptors [62] or inhibition of plasma-membrane Ca^2+^-ATPase (PMCA) [68].

Taken together, our data from Ca^2+^ functional imaging demonstrates that:(i) acute treatment with low, sublethal doses of extracellular-applied NH_2_htau is not able to modify the resting Ca^2+^ level but affects both the magnitude and the kinetic parameters of ionic intracellular signaling evoked by chemically-induced depolarization of the synaptic neuronal compartments; (ii), the amplitude of K^+^-evoked Ca^2+^ transient is greater in synaptosomes when exposed to NH_2_htau but declines to a stable plateau more quickly (i.e higher and shorter signal) than in the other two experimental groups (reverse- and saline-treated controls); (iii) this effect is specific because its reverse sequence is actually inactive in perturbing either basal or stimulated Ca^2+^ homeostasis when compared to saline-exposed untreated controls.

### Extracellular-added NH_2_htau acutely inhibits K^+^-induced FM1-43 destaining and presynaptic glutamate release in hippocampal synaptosomal preparations

**Figure 4:**
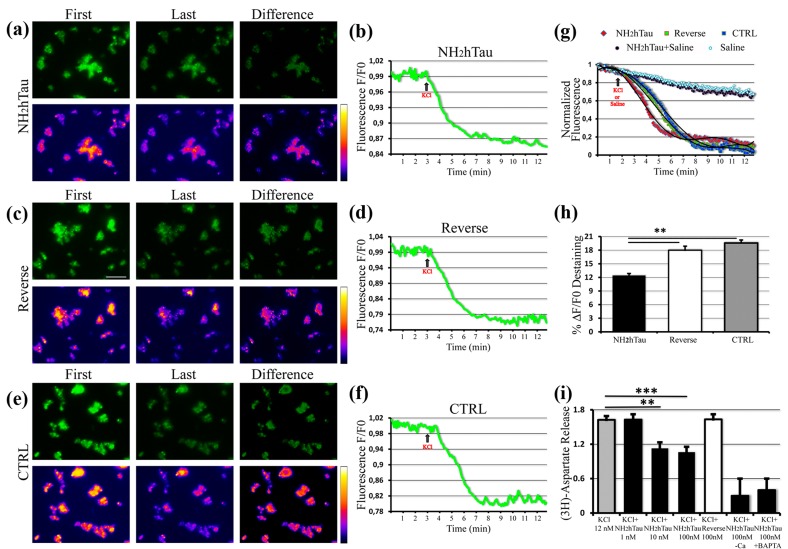
NH_2_htau acutely inhibits the K^+^-stimulated presynaptic vesicles release and glutamate exocytosis in purified synptosomal preparations **a.**-**b.**-**c.**-**d.**-**e.**-**f.**-**g.**-**h.** K^+^-induced destaining of FM1-43 dye on isolated nerve terminals from mature (15 DIV) hippocampal neurons exposed to NH_2_htau (1µM), its reverse peptide (1µM), and saline (CTRL) 5 min before KCl (30mM) stimulus addition. **a.**-**c.**-**e.** Upper row: wide field images of treated synaptosomes. Lower row: false color vertically corresponding images (fire palette) which show the fluorescence intensity. Brighter spots correspond to functional presynaptic terminals. First is the image before KCl administration. Last is the image when the after-stimulus baseline is established. Difference is subtraction of the last image from the first one. Note that the yellow color is below the saturation level (i.e.white color). **b.**-**d.**-**f.** Representative traces of destaining time courses derived from the three experimental groups. **g.** Normalized, aligned and averaged fluorescence intensity traces derived from the three experimental groups (25 traces each) plus two saline(-KCl) additional controls. One, in the absence of both treatments and KCl stimulus, representing the fluorescence bleaching rate of our experimental setting. The other one, in the presence of NH_2_htau (1µM) and with saline added, showing that NH_2_htau alone is not able to induce significant destaining effects. Trend lines (black lines) superimposed to fluorescence intensity values of three experimental groups were calculated by polynomial fitting. **h.** Comparison of the average destaining percentage of the fluorescence intensity among the three experimental groups. In each experiment(n=5) two coverslips for each experimental group were analyzed. Values are means of at least three independent recordings and statistically significant differences were by one-way ANOVA followed by Bonferroni post-hoc test (**p<0,01versus NH_2_htau). Scale bar:15µm. **i.** The overall dose-effect of the NH_2_htau action (1-10-100nM) on glutamate release was evaluated by high-sensitive radioactive-based measure of depolarization-evoked release of [3H]D-Asp. Reverse sequence, used at the highest concentration(100nM) and saline-exposed untreated controls were also included. The K^+^-evoked tritium overlow is expressed as % of 12 mM KCl-evoked [3H]D-aspartate overflow versus saline-exposed untreated ctrl.Values are means of at least five independent experiments and data were considered statistically significant for p < 0.05 at least (**p < 0,01; ***p < 0,0001 versus saline-exposed untreated ctrl, one-way ANOVA followed by Bonferroni post-hoc test).

Owing to potent relationship between Ca^2+^ and exocytosis, small changes in Ca^2+^ influx during a presynaptic potential action are highly effective in modulating the neurotransmitter output at synapses [69]. Glutamate is the primary excitatory neurotransmitter in the brain playing an important role in hippocampal-dependent learning and memory and deregulation of glutamatergic synaptic transmission is considered a primary step of synaptic failure occurring in the pathogenesis of AD [70]. In view that our results showing an early modulatory effect of NH_2_htau on K^+^-evoked regulation of intracellular Ca^2+^ (Figure 3) hinted at possible alterations in Ca^2+^-dependent mechanisms underlying synaptic activity, we evaluated the acute effects evoked by the extracellular addition of either NH_2_htau and its reverse sequence (1 µM each) on live dynamics of vesicles release from isolated nerve terminals (i.e. synaptosomes) of mature (15 DIV) hippocampal neurons by means of functional FM1-43 fluorescence imaging. FM1-43 styryl dye is more fluorescent when partitioned in membranes, so its release from synaptic vesicles can be measured as a monoexponential loss in signal which actually reflects the rate of exocytosis occurring at nerves terminals. Although this protocol is unable to discriminate glutamatergic from GABAergic synapses, its activity-dependent unloading is widely used to study the properties of presynaptic release on purified synaptosomal preparations, providing thus a direct measure of their *in vitro* secretory function [59, 71, 72, 73]. After 5 min exposure to NH_2_htau, its reverse sequence and saline, the decay of FM1-43 fluorescence evoked by synaptosomes stimulation with high-potassium (30 mM KCl) was measured and quantitative analysis of live imaging data from the three different experimental groups is shown in Figure 4a-4b-4c-4d-4e-4f-4g-4h. Results clearly indicate that short-term application of subtoxic doses of extracellular NH_2_htau caused a significant reduction of pre-synaptic FM1-43 destaining (NH_2_htau: 12, 42 %+/- 4, 7 *n* = 90, one-way repeated-measures ANOVA followed by Bonferroni post-hoc test **p < 0, 01) in individual hippocampal axonal terminals which displayed severe deficits in vesicles release dynamics when compared to both reverse- and untreated controls (-36, 56 % vs ctrl; -30, 94 % vs reverse). Conversely, reverse peptide did not provoke any appreciable change in activity-dependent dye unloading (reverse: 17, 98 % +/- 6, 1 *n* = 86) and reverse-treated nerve terminal preparations did not significantly differ (-8%) from untreated controls (ctrl: 19, 58% +/- 3, 2 *n* = 90, one-way repeated-measures ANOVA followed by Bonferroni post-hoc test p >0, 05). Of note, when hippocampal synaptosomes were exposed to extracellular NH_2_htau used at the same experimental concentration (1 µM) but in the absence of KCl stimulus (resting conditions, saline-exposed), we did not detect significant differences (Figure 4g) in comparison to an untreated and saline-exposed group ruling out thus the possibility that this extracellular-added peptide might have a destaining effect *per se* .

To further investigate the nature of potent *in vitro* inhibitory effect shown by NH_2_htau on presynaptic compartment of hippocampal neurons, high-sensitive radioactivity-based assays of glutamate release were also carried out according to standard procedure [74, 75]. To this aim, isolated nerve endings preloaded with [3H]D-aspartate ([3H]D-Asp) -which allows to measure the neurotransmitter exocytosis avoiding drawbacks due to its enzymatic degradation- were incubated with synthetic NH_2_htau or its reverse counterpart (1, 10, 100nM) and then superfused with mild depolarizing stimulus (12 mM KCl; equiosmolar replacement of Na^+^) in order to elicit the Ca^2+-^dependent, exocytotic-like release of the radioactive tracer [76, 77, 78]. As shown in Figure 4i, transient and short-term exposure (-90 s) of synaptosomal terminals to nanomolar concentrations of NH_2_htau caused a dramatic and statistically significant inhibition of 12 mM K^+^-evoked tritium overflow in a concentration-dependent manner, the maximum of inhibition (-38, 3%) being observed when the peptide was added at 100 nM (NH_2_htau: 1 +/- 0, 09 *n* = 11 ANOVA followed by Bonferroni post-hoc test ***p < 0, 0001 vs ctrl). On the contrary, reverse peptide even if used at the highest experimental concentration (100 nM) was completely inactive in modulating the exocytotic-like glutamate release (reverse: 1, 63 +/-0, 09 *n* = 11) and treated samples did not significantly differ from saline-exposed untreated controls (ctrl 1, 62 +/- 0, 06 *n* = 11).

No significant effect on spontaneous basal glutamate release was detected in isolated synaptosomes upon long-term incubation with NH_2_htau and its effect on K+ evoked neurotransmitter exocytosis was abrogated by incubation in Ca^2+^-free milieu or by pretreatment with membrane-permeant ion chelator 1, 2-bis-(2-aminophenoxy)ethane-N, N, N’, N’-tetraacetic acid tetra(acetoxymethyl) ester (BAPTA/AM)BAPTA, suggesting that the presynaptic action of NH_2_htau is strictly Ca^2+^- dependent (Figure 4i).

Importantly, these functional changes at synaptic terminals occurred within few minutes of exposure, in agreement with prompt and marked tendency of NH_2_htau to interact with biomimetic and cell-surface membranes as we previously detected by morphological, biochemical and biophysical studies (Figure 2). Besides, the extracellular-added NH_2_htau, but not its reverse counterpart, was able to alter the K^+^- stimulated functional properties in neurotransmitter secretion of hippocampal neurons in the absence of any appreciable cell injury because the loss in synaptic components along with the structural changes in neuritic architecture and the reduction in mitochondrial density were measured only later, after 48h incubation times (see below) .

Collectively these experiments, carried out on isolated hippocampal synaptosomes by Ca^2+^-indicator dye measures and neurotransmitter exocytosis assays, points to a direct causal link between depolarization-dependent defects in Ca^2+^ handling and early presynaptic modulatory effect of extracellular NH_2_htau on glutamatergic neurotransmission demonstrating that: (i) the inhibitory action on depolarization-stimulated glutamate exocytosis induced by short-term external application of low, sublethal doses of NH_2_htau is accompanied by concomitant alteration in local Ca^2+^ dynamics in nerve endings; (ii) the peak amplitude and kinetic parameters of Ca^2+^current evoked by K^+^ stimulation develop respectively at a greater rate but decay to a less long-lasting plateau in NH_2_htau-exposed in nerve endings compared with control ones (i.e higher and shorter signal), resulting in an overall decrease in neurotransmitter release ; (ii) these changes are specific for NH_2_htau because its reverse sequence does not afford any significant effect either on stimulated Ca^2+^ signaling or on glutamate exocytosis when compared to saline-exposed untreated controls.

### Deterioration in presynaptic terminals, neuritic degeneration, microtubule collapse and reduction of mitochondrial density are detected in living hippocampal neurons only after long-term exposure to sublethal doses of NH_2_htau

**Figure 5:**
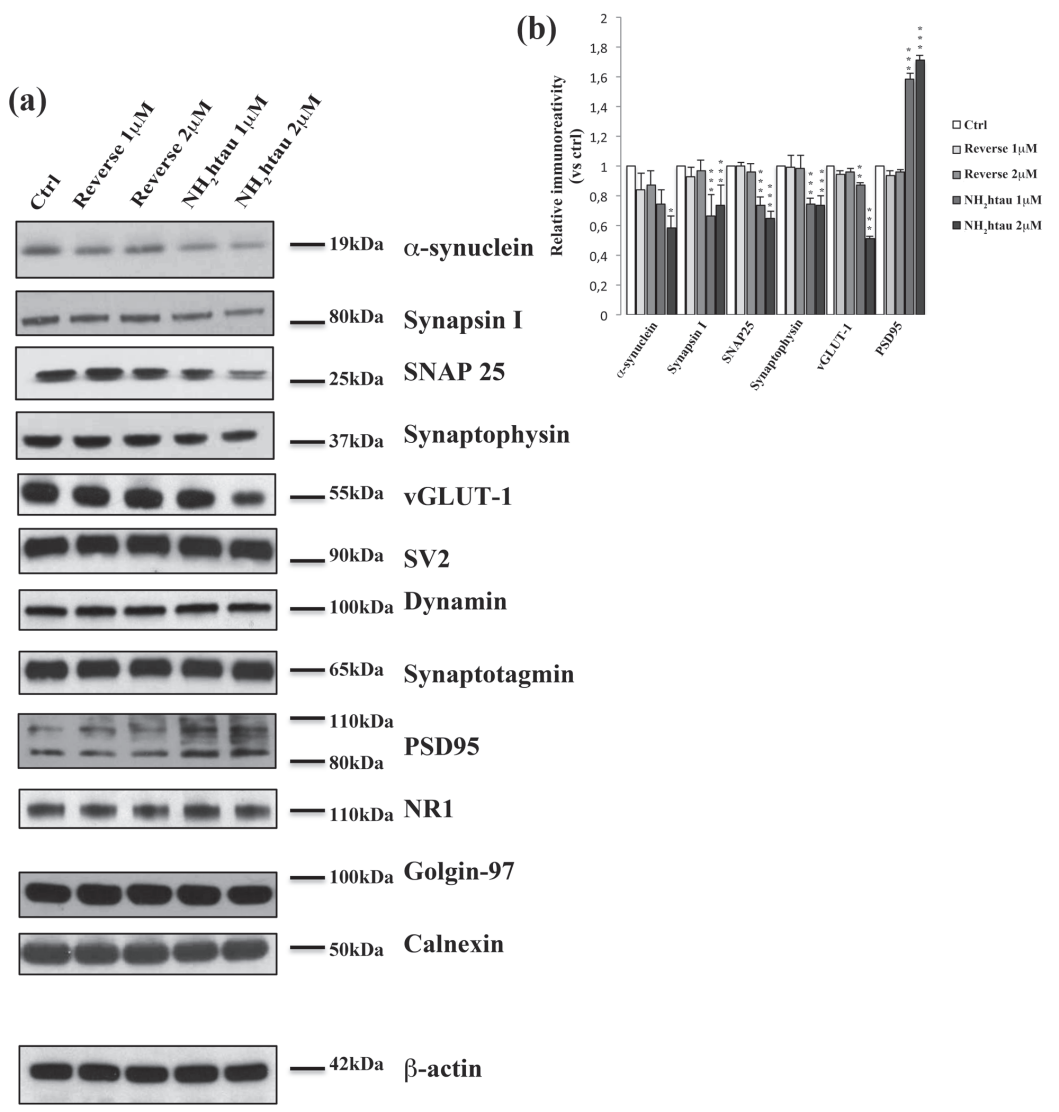
Long-term application of NH_2_htau induces a marked and selective loss of exocytotic presynaptic vesicles proteins in cultured hippocampal neurons **a.**-**b.** Western blotting analysis (n=12) was carried out on equal amounts of total protein extract (40µg) from mature hippocampal primary neurons (DIV15) exposed for 48h to increasing subtoxic concentration (1-2µM) of NH_2_htau and its reverse control sequence. Immunoblots (**a**) were probed with antibodies against several presynaptic- (α-synuclein, synapsin I, synaptosomal-associated protein 25 SNAP-25, synaptophysin, vesicular glutamate transporter 1 vGLUT1 , synaptic vesicle protein 2 SV2, dynamin, synaptotagmin) and post-synaptic markers (N-Methyl-D-aspartate NMDA Receptor Subunit NR1, postsynaptic density protein 95 PSD95) and against not-synaptic proteins located in trans-Golgi network and endoplasmic reticulum (golgin-97 and calnexin). Cropped representative WB are shown. Densitometric quantification of immunoreactivity levels (**b**) was calculated by normalizing the values on the β-actin intensity and expressed as ratio respect to corresponding ctrl values.Values are means of at least nine independent experiments and statistically significant differences were calculated by one-way ANOVA followed by Bonferroni post-hoc test (*p < 0,05; **p<0,01; ***p<0,0001 versus untreated ctrl).

Structural alterations in synaptic terminals, instability of microtubules, dendritic retraction, mitochondrial abnormalities are all hallmarks of neuronal pathology playing a critical role in promoting cognitive dysfunction during the AD onset/progression and appearing prior to frank cell death [79]. Therefore, by Western blotting on total protein extracts and immunocytochemistry analyses, we investigated the expression level and subcellular distribution of different cellular markers of synaptic, cytoskeleton and mitochondrial compartments after administration of NH_2_htau (1-2µM) to hippocampal neuronal cultures for increasing incubation times (up to 48-72 h). Interestingly (Figure 5a-5b), we noticed only after 48h cultures treatment a significant and dose-dependent decline in selected proteins which are mainly located in the presynaptic compartment and are involved in local turnover of synaptic vesicles and/or Ca^2+^-triggered neurotransmitter release, such as α-synuclein, synapsin I, synaptosomal-associated protein 25 (SNAP-25), synaptophysin and vesicular glutamate transporter 1(vGLUT1). Importantly, no discernable change was detected at earlier incubation times (1-3-6-12-24h, data not shown) ruling out the possibility that the diminution in functional release of glutamate induced by the acute administration of NH_2_htau on isolated hippocampal synaptosomes -as we showed in Figure 4- could be ascribed to modifications in expression levels of these relevant presynaptic proteins which control the Ca^2+^-coupled neurotransmitter exocytosis at nerve endings. Others presynaptic proteins -such as synaptic vesicle protein 2 (SV2), dynamin, synaptotagmin- were unmodified, in line with the *in vivo* evidence that presynaptic proteins are not equally affected in AD brains [80, 81, 82]. Conversely, the expression level of two major postsynaptic proteins -N-Methyl-D-aspartate (NMDA) Receptor subunit NR1 and postsynaptic density protein 95 (PSD95) - did not change or even significantly increased, likely due to reactive/compensatory mechanisms reminiscent of those occurring *in vivo* during progression of AD pathology [83, 84]. Other postsynaptic proteins, such as NMDAR2A/B and α-amino-3-hydroxy-5-methyl-4-isoxazolepropionic acid (AMPA) Receptor subunit GluR1/5, were also unaffected throughout our experimental conditions (data not shown). No significant alterations in the intracellular amount of not-synaptic proteins located in different subcellular compartments- including specific markers of trans-Golgi network and endoplasmic reticulum such as golgin-97 and calnexin- were contextually found.

**Figure 6:**
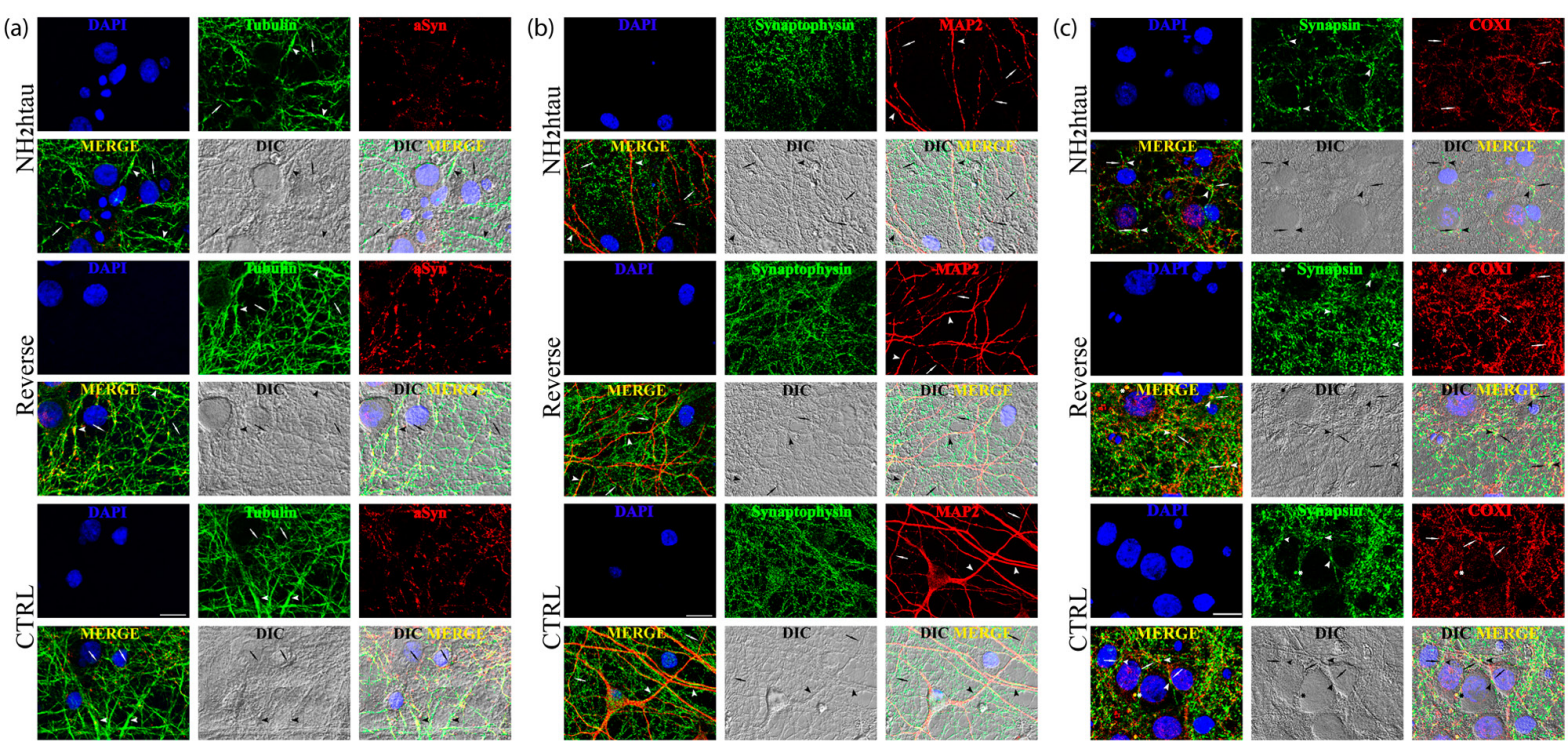
Distortion of the dendritic tree, microtubule breakdown and mitochondria loss occur in concomitance with decline of presynaptic proteins density in living hippocampal neurons chronically exposed to NH_2_htau **a.**-**b.**-**c.** Confocal microscopy analysis of double immunofluorescence carried out on mature hippocampal primary neurons (DIV15) exposed for 48h to NH_2_htau and its reverse control sequence (1µM). Merge images show the overlay of the three fluorescence channels, Differential Interference Contrast (DIC; gray channel) enables the visualization of the neuritic network, DIC Merge is the composition of the three fluorescence and of the DIC channels. (a): presynaptic synaptophysin (green channel) and dendritic MAP-2 (red channel). Nuclei (blue) were stained with Hoechst 33258 (0.5 mg/ml). Arrowheads and arrows point to MAP2- positive neurites of larger and smaller caliber, respectively. (b): presynaptic α-synuclein (green channel) and neuron-specific cytoskeletal beta III tubulin (red channel). Arrowheads and arrows point to beta III tubulin-positive neurites of larger and smaller caliber, respectively. (c): presynaptic synapsin I (green channel) and mitochondrial marker COX I (red channel). Arrowheads point to synapsin I-labeled presynaptic spots and arrows point to COX I -positive mitochondrial structures. In the merge, DIC and DIC-Merge channels, arrowheads and arrows appear in opposition to give evidence to mitochondria resident at juxstaposed presynaptic sites. Asterisks mark typical punctuate structures immunoreactive for both synapsin I and COX I (yellow dots) representing mitochondria which are localized to presynaptic sites (i.e.synaptic mitochondria). Note the loss of double-stained synapsin I/COX I puncta and the decrease of juxstaposed presynaptic sites/mitochondria in the NH_2_htau-treated cultures. Images are representative of at least three independent experiments. Scale bar: A=20 µm ;B-C=10 µm.

Furthermore morphological quantitative studies (Figure 6a-6b-6c-Supplementary Figure 4), in addition to confirming the loss of selective pre-synaptic proteins detected in above-mentioned biochemical analyses, revealed that a concomitant disruption of cytoskeleton took place in NH_2_htau-treated hippocampal cultures which displayed a simplification in network of neuronal processes along with an evident corruption/diminution in array of microtubule track (length and assembly). A pronounced decrease in immunoreactivity of MAP-2, a microtubule-associated protein largely used to trace the integrity of the neuritic network, was clearly evident in NH_2_htau-treated neuronal cultures when compared to control and reverse groups (one-way repeated-measures ANOVA followed by Bonferroni post-hoc test ***p <0, 0001) in concomitance with a marked drop in the density of puncta positive for synaptophysin, a specific marker for presynaptic membrane vesicles (one-way repeated-measures ANOVA followed by Bonferroni post-hoc test ***p <0, 0001) (Figure 6a, Supplementary Figure 4C-4D). The loss in MAP-2 staining turned out to be mostly localized to neurites of thin calibers (Figure 6A, arrows) which appeared distorted, fragmented and decorated with interspersed bead-like varicosities whereas the larger processes (Figure 6a, arrowheads) were still present and appeared uninjured. Moreover, after staining of neuron-specific βIII-tubulin, NH_2_htau-treated neuronal cultures showed in comparison to control and reverse groups an evident neuritic dystrophy (one-way repeated-measures ANOVA followed by Bonferroni post-hoc test ***p <0, 0001) involving both high and low calibers processes (Figure 6b, arrows and arrowheads respectively, Supplementary Figure 4A). Destabilization and reduction in length of cellular microtubules occurred in concomitance to a net decrease in dotted labeling for α-synuclein(one-way repeated-measures ANOVA followed by Bonferroni post-hoc test ***p <0, 0001, Supplementary Figure 4B), a presynaptic protein which is known to be also severely affected in several neurodegenerative tauopathies including AD [85]. Besides, a significant reduction in density of COX-1- positive mitochondria was also clearly appreciable in NH_2_htau-exposed neuronal cultures (one-way repeated-measures ANOVA followed by Bonferroni post-hoc test ***p <0, 0001, Supplementary Figure 4E-4F), just resembling the retrograde degeneration or “dying-back neuronal death” as detectable at prodromal AD stages [86, 87, 88]. In particular, mitochondria co-located (Figure 6c, asterisks in reverse-treated and control groups) or juxtaposed to synaptic sites (Figure 6c, arrowheads and arrows in opposition) appeared mainly affected in primary cultures upon long-term treatment with extracellular NH_2_htau. In line with previous findings reporting a causal relationship between tau malfunction, depletion of synaptic mitochondria and loss of synaptic markers [89], the impaired trafficking of these organelles which were likely no longer transported along compromised axonal projections towards terminal ends [90] occurred along with progressive and delayed *in vitro* presynaptic deterioration, as displayed by contextual decline in synapsin-I immunoreactivity. Importantly, in spite of these neuritic and synaptic degenerative alterations, only very few neurons displayed an evident nuclear heterochromatin, indicating that most of these pathological changes occurred before an overt neuronal death (Supplementary Figure 3). Finally, no changes in global cellular integrity/viability (Supplementary Figure 3) were found in untreated and reverse-treated controls used at the same experimental conditions (concentration and time incubation), although we detected a slight variability in the latter experimental group in agreement with previous findings reporting a quantifiable but not statistically-significant interfering response in hippocampal neurons exposed to other backward-reading sequence peptides such as the reverse Aβ peptide [91].

Taken together, these studies suggest that: (i) chronic exposure of mature hippocampal primary neurons to low and sublethal doses of extracellular NH_2_htau recapitulates important features of *in vivo* pre-symptomatic stages of AD neuropathology resembling the dying-back mechanisms of cell degeneration ; (ii) NH_2_htau -but not its reverse sequence- is able to specifically interfere with structural stability of presynaptic terminals only after chronic treatment of cultures (48-72h) in concomitance with pronounced perturbations in number of resident mitochondria and/or cytoskeleton organization.

### NH_2_htau peptide shows a higher propensity to assume less compacted conformers than its reverse counterpart: conformational flexibility may account for their divergent *in vitro* biological effects

Proteolytic cleavage of tau alters its structure, functional capacity and propensity to aggregation [1]. Nevertheless, given their intrinsically flexible nature, tau and its truncated forms do not retain a fixed 3D structure but fluctuate among a large number of different configurations and, consequently, relative structural studies turn out to be problematic and cannot be performed by means of standard techniques, such as X-ray diffraction and electron microscopy [92, 93, 94, 95]. In this context, small angle X-ray scattering (SAXS) is one of the few biophysic approaches which is able to provide detailed structural information on this protein, allowing a quantitative characterization of its conformational polydispersity [93, 94, 95]. Accordingly, the SAXS method has been widely employed in recent years to study the overall structure of both full length tau and its truncated forms [95, 96, 97, 98, 99, 100, 101] and also to characterize other neuropathologically-relevant small peptides composed of only few tens of residues such as the neurotoxic Aβ1-42 (42 aminoacids) [102] and the chemically unfolded Angiotensin II (8 aminoacids) [103]. However, at variance with C-terminal fragments that have been extensively studied [95, 99, 104], very little is known about NH_2_-terminal fragments of tau protein whose structural characterization can provide a better understanding of their potent neurotoxic role [13, 33, 34, 105].

**Figure 7:**
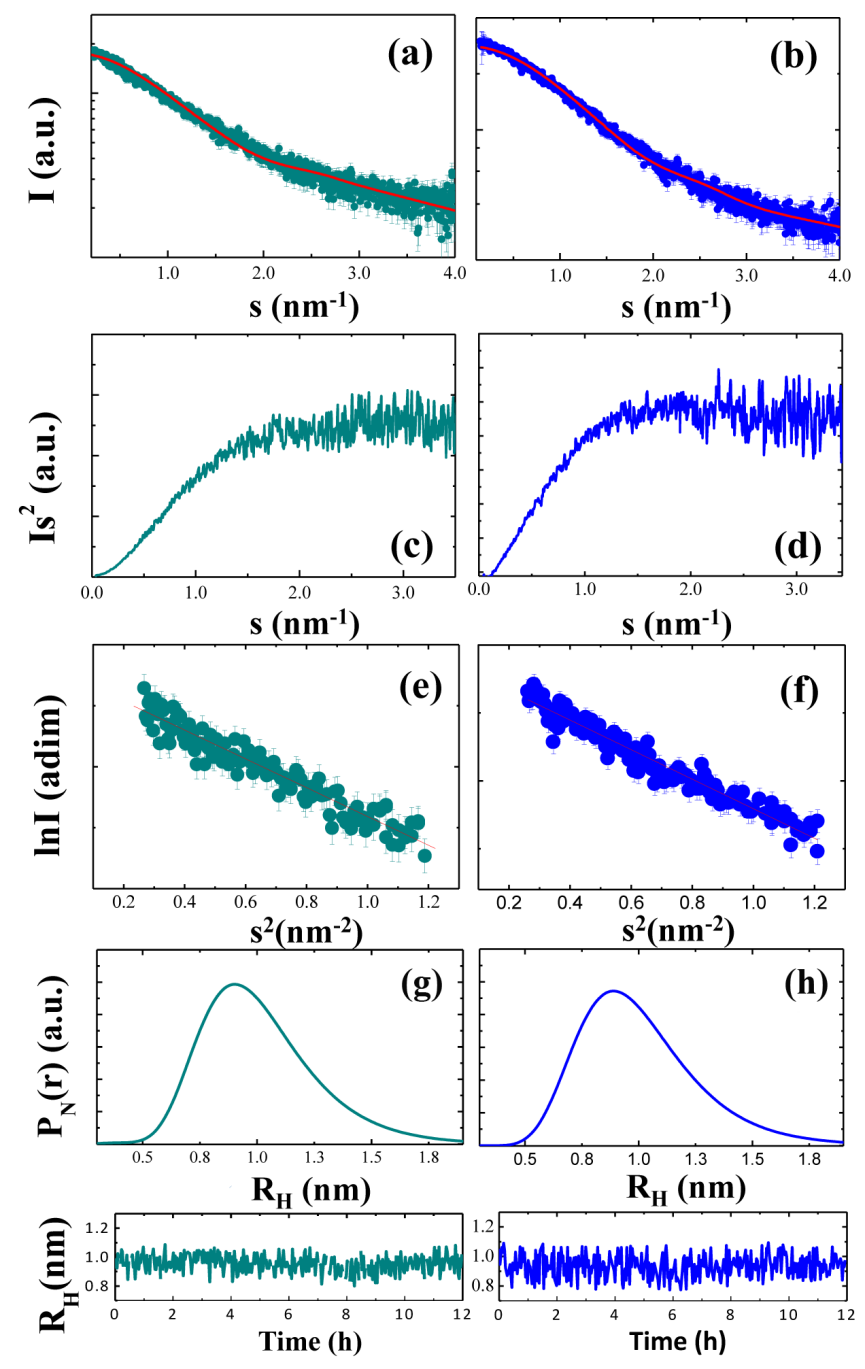
NH_2_htau is more extended than its reverse counterpart, hinting at different conformational flexibility From top to bottom of the panel are reported: scattering profiles of NH_2_htau (**a**) and its reverse sequence (**b**) and the red continuous lines indicate the EOM fit of the experimental data; Kratky plots of the two peptides (**c**, **d** for NH_2_htau and its reverse control sequence, respectively); Guinier plot of the two peptides (**e**, **f** for NH_2_htau (a) and its reverse sequence, respectively); Number-weighted hydrodynamic radius distributions P_N_(r) (**g**, **h** for NH_2_htau and its reverse control sequence, respectively); time evolution of the average number-weighted hydrodynamic radius (lower panels of fig g-h for NH_2_htau and its reverse sequence, respectively).

Here, we used SAXS to investigate the overall conformation of the NH_2_htau and its reverse sequence peptide. Figure 7a-7b displays the processed scattering curves I(s) of two measured tau constructs with both profiles appearing featureless, as expected for a flexible and unstructured peptide. Their flexible nature was also confirmed by the Kratky plot (s^2^I(s) as a function of s) which is traditionally employed to qualitatively identify disordered protein states (Figure 7c-7d, for NH_2_htau and its reverse respectively ) [92, 93, 95, 97, 98, 99, 106]. As expected, Kratky plots (Figure 7c-7d) were not bell-shaped with a clearly defined maximum. For both tau peptides, s^2^I(s) monotonously increased up to reach a plateau at high s values -a behavior which resembleed that of an ideal Gaussian chain [106]- further confirming their unstructured nature. The gyration radius R_G_ of NH_2_htau and its reverse peptide were then estimated by using the Guinier approximation -according to which the scattering intensity has a Gaussian shape at small s (s<1.3/R_G_) [92, 93, 94, 95, 97, 98, 99, 106]- and the calculated Guinier plot (lnI(s) vs s^2^) is reported in Figure 7e-7f respectively. As shown by a linear fit of the data, we obtained R_G_ = 1.32±0.02 nm for NH_2_htau26-44 and 1.23±0.02 nm for its reverse sequence peptide. Similar results were found by using the Debye’s approximation (data not shown) and the R_G_ values are summarized in Table 2.

**Table 2:** Peptides concentration as measured by using the Guinier’s approximation (first column); gyration radius R_G_ (second column) and hydrodynamic radius (third column) as measured by SAXS and DLS; R_G_/R_H_ ratio obtained by columns 2 and 3 (4th column); Calculated R_G_ value for an ideal Gaussian Chain according to eq. 1 (5th coulmn).

Peptide	Concentration mg/ml	R_G_ (nm) Guinier	R_H_ (nm) DLS	R_G_ /R_H_	R_G_ (nm) R_G_ =R_0_N^ν^	R_G_ (nm) EOM	A_0.155_/A_0.97_
**NH**_2_**htau**	2.5±0.1	1.32±0.03	0.95±0.05	1.4±0.1	R_G_=1.18±0.06	1.31±0.05	3.1±0.2
**Reverse**	2.4±0.1	1.23±0.03	0.94±0.05	1.3±0.1	R_G_=1.18±0.06	1.21±0.05	1.5±0.2

**Figure 8:**
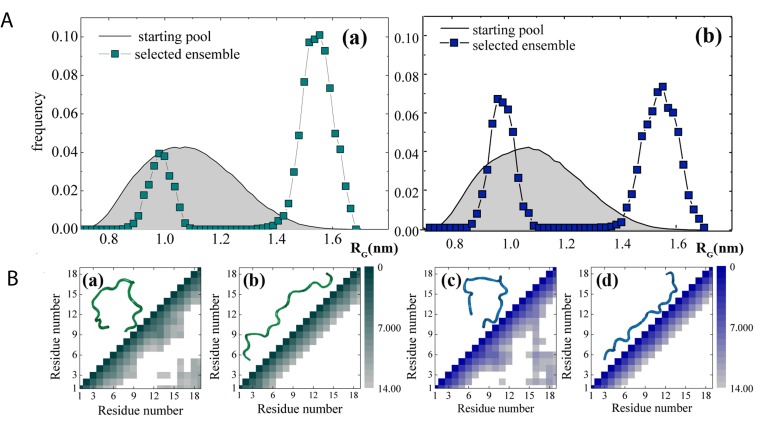
Ensembles of structures populated by NH_2_htau in aqueous solution differ from those by its reverse sequence **A.** EOM starting pool of conformations (gray shaded curves) and selected ensembles (filled square) for NH_2_htau (**a**) and its reverse counterpart (**b**). **B.** contact maps (C_α_–C_α_ distance between all pairs of residues) of four highly probable conformations, namely: a highly probable NH_2_htau/reverse sequence conformation extracted from the peak at low R_G_ values (**a**-**c**) and high R_G_ value (**b**-**d**). Distances between different residues are expressed in Å (a cut-off distance at 14 Å was used). In the figure insets, a snapshot of the corresponding configuration is reported.

R_G_ value computed by using the Ensable Optimization Method EOM (6th coulmn); A_1.5_/A_1_ values obtained by dividing the area of the peak at high R_G_ by that of the peak at low R_G_ value in Figure 8A.

An important metric holding for unstructured proteins and peptides is given by the Flory equation that correlates R_G_ with the length of the peptides [103]:

$$R_{G}=R_{0}N^{v}$$ (eq. 1)

where N is the number of aminoacids, R_0_ is a constant that depends on the persistence length of the chain and ν = 1/2 for a Gaussian chain. For R_0_ = 2.54±0.01 nm and v = 0.598±0.028, Eq. 1 well describes the behavior of unfolded proteins and peptides ranging between 16 and 549 residues [103]. By using eq. 1, a theoretical value of R_G_ = 1.18±0.03 nm could be estimated for both peptides and this value was consistent within one standard deviation with that we measured for the reverse sequence, again endorsing its flexible and disordered nature. Conversely, NH_2_htau showed a slightly higher R_G_ than that expected for a Gaussian chain, being consistent with the theoretical values within three standard errors.

An independent experimental approach to probe the flexible nature of the two peptides was obtained by comparing the measured R_G_ with the average hydrodynamic radius R_H_ [107, 108]. In view of these considerations, we measured the hydrodynamic radius of NH_2_htau and its reverse sequence by dynamic light scattering (DLS), at the same temperature and concentration conditions used in SAXS experiment. In Figure 7 we report the number-weighted hydrodynamic radius distributions (P_N_ (r) ) of NH_2_htau (Figure 7g) and its reverse sequence (Figure 7h) which were computed by averaging a few hundreds of independent P_N_(r) functions. As shown (Figure 7g-7h), distributions were monodisperse confirming that the two peptides were of high purity and did not contain significant amounts of aggregates. An average number-weighted hydrodynamic radius (R_H_ )_N_ = 0.95±0.05 nm (eq. 4) and (R_H_ )_N_ = 0.94±0.05 nm were measured for NH_2_htau and its reverse sequence, respectively. The stability of the two peptides was also assessed by monitoring (R_H_ )_N_ over a time of 12 hours (Figure 7g-7h lower panels). Interestingly and in agreement with data shown in Figure 1f, (R_H_ )_N_ remained constant during 12 hr incubation confirming thus that the large part of species in solution was in monomeric form.

A R_G_/R_H_ ratio of 1.4±0.1 and 1.3±0.1 was obtained for NH_2_htau and its reverse sequence, respectively. As reported, these values were consistent with the value for an ideal Gaussian chain as expected for a highly denaturated protein or an intrinsically disordered protein in their Θ-state [107]. At the Θ-point*, chain-chain and chain-solvent interactions balanced each other such that the polymer was at a critical point, at which the thermodynamic phase boundaries disappeared. Collectively, these biophysical data further corroborate the highly flexible nature of the two peptides and are in close agreement with our CD experiments showing that the only structure detected in a wide pH range (pH 4-11) is random coil (Figure 1a).

To gain better insights into the overall structure of the two analyzed peptides, we fitted their experimental curves by using the Ensemble Optimization Methods (EOM) implemented in the software package ATSAS [92, 93, 94, 95], which has been widely used to characterize many intrinsically disordered proteins, including the full length tau and its truncated forms [95, 97, 98, 99] and the neurotoxic Aβ 1-42 peptide [102]. The EOM method -starting from the aminoacidic sequence- generates a pool of models spanning the entire chain’s conformational space and then it selects from that pool -by an iterative genetic algorithm- the most probable ensemble of conformations which best fit the experimental data. The R_G_ distributions of the starting pool of conformations (gray shaded curve) as well the selected ensemble (open square curve) for both NH_2_htau and its reverse sequence peptide are reported in Figure 8A (a-b, respectively). As shown, the selected ensembles matched the experimental curves (Figure 7a-7b; continuous red lines). Notably, in both cases, the selected ensembles did not span the entire available conformational space but only two well-defined regions peaked around 1 and 1.5 nm, a behavior which was confirmed by generating four independent starting pools of different numerosity (Supplementary Figure 5). The selected ensembles clearly demonstrated that the reverse sequence peptide had a higher tendency to populate more compacted conformational states in comparison to NH_2_htau (Figure 8A a-b) and, relevantly, this propensity was consistent with the higher R_G_ value of NH_2_htau obtained in Figure 7e-7f. A quantitative estimation of this trend can be provided by the A_1, 5_/A_1_ ratio, a parameter which was obtained by dividing the area of the peak at high R_G_ by that of the peak at low R_G_ value. To this point, we measured a value of A_1, 5_/A_1_ = 3.2±0.2 for NH_2_htau- and of A_1, 5_/A_1_ = 1.5±0.2 for its reverse counterpart (errors correspond to the weighted standard deviation calculated over the four different ensembles reported in Supplementary Figure 5). Importantly, the A_1, 5_/A_1_ ratio was found to be significantly different between the two analyzed peptides, further supporting the different sampling of the phase space which may underlie their different biological *in vitro* effect. Remarkably, it has been largely accepted that the pathogenic role of unstructured proteins and peptides cannot be related to misfolding of given fixed 3D structures, as in the case of globular protein, but it is more likely to be strictly correlated to the their different capability to fluctuate among different conformations which can be significantly altered in pathological conditions [43] .

Finally, Figure 8B displays four contact maps (C_α_-C_α_ distance expressed in Ångström between all pairs of residues) of four highly probable conformations, namely: a highly probable NH_2_htau/reverse sequence conformation extracted from the peak at low R_G_ values (Figure 8B a-c) and high R_G_ values (Figure 8B b-d). A cut-off distance of 14 Å was used in these analyses. In Figure 8B a-c, we highlighted the occurrence of a long-range contact between the region entailing residues 15−19 and the region entailing residues 1−5. Furthermore, in the figure insets, a snapshot of the corresponding configuration is reported. It is worth stressing here that these conformers are not meant to provide a statistically significant description of the overall conformations of peptide. Conversely, they provide an idea of what might be the shape of a compacted and an extended conformer.

Altogether and in agreement with CD spectra and ThT fluorescence intensity measurements (Figure 1a-1b-1c-1d-1e), our SAXS analyses confirm that NH_2_htau clearly displays a disordered and monodispersed nature under the current experimental conditions. Furthermore, these results demonstrate that -in contrast to its reverse sequence counterpart- the NH_2_htau is more prone to adopt a specific subsets of “open” conformations - likely by exposing important surface residues essential for its functional *in vivo* interaction with specific biological partners- which might account for their selective and divergent biological effects. Finally, the present data will be in aid of future immunotherapeutic interventions [21] providing new structural information on the 18-residue domain located in N-terminal projection of human tau (26-44epitope) which is the minimal biologically active moiety of longer secreted 20-22kDa pathologic peptide [27, 34] .

## DISCUSSION

Truncation at N-terminal domain of tau plays an important role in both neurodegeneration and cognitive decline occurring in all brain human tauopathies, including the most prevalent Alzheimer’s disease (AD) [109]. Tau cleavage has been largely accepted to critically contribute to pathogenesis and progression of these devastating disorders not only by promoting misfolding /aggregation of protein but also by releasing soluble toxic fragments which induce neurodegeneration in a way independent of aggregation [110). Considered that: (i) a better understanding of early, asymptomatic and possible reversible states in progression of neuropathology of these illnesses could help to plan preventive and then more effective disease-modifying therapeutic strategies [111]; (ii) the extension of tau neuropathology more closely correlates with dementia status, being a better predictor of cognitive performance than Aβ deposition in any region of the brain [112]; (iii) immunotherapy-based *in vivo* Aβ reduction has been proved to have only a limited success [4, 5] it has been suggested that a greater clinical efficacy may be achieved by clearing extracellular, soluble toxic tau species in the earlier stages of the disease when cognitive impairments is still not evident [5]. In this regards, passive immunization targeting the N-terminally truncated forms of tau is being currently pursued in phase I clinical trials [113] based on the findings that multiple fragments containing NH_2_/mid-region of human protein -but not its C-terminally cleaved and full length isoform(s)- are mostly detected in CSF from AD patients [9, 10, 11, 12] and in conditioned media from AD patient-derived induced pluripotent stem cells (iPSC) cortical neurons [13, 14].

Here we show that low, sublethal doses of soluble and extracellular-added human NH_2_tau 26-44 fragment (i.e.NH_2_htau) - which is the minimal biologically active moiety of neurotoxic 20-22kDa parental peptide accumulating *in vivo* in AD presynaptic terminals and secreted into extracellular parenchyma [24, 25, 28, 33, 34]- are able to impact on normal synaptic function(s) by acutely interfering with depolarization-evoked glutamate release from purified hippocampal nerve terminals. Significant reduction in presynaptic neurotransmitter exocytosis is paralleled by local alteration in peak amplitude and kinetic parameters of Ca^2+^ transients which concomitantly take place in K^+^-stimulated isolated synaptosomal preparations. Biochemical and morphological changes, such as selective reduction in presynaptic proteins along with marked neuritic dystrophy and loss of synaptic mitochondria which are classically detected in the pre-symptomatic neuropathologic stages of human tauopathies, are found in *in vitro* neuronal cultures only after chronic incubation with extracellular NH_2_htau and prior to overt cell death. The specificity of these results is further supported by the lack of any significant biological effect contextually shown by application of reverse peptide which behaves as inactive control in all the above-summarized analyses, likely due to its poor conformational flexibility which makes it unable to dynamically perturb lipid membranes in contrast to NH_2_htau. This evidence is biologically relevant to human pathology and important in terms of translational outcome, in view the fact that a population of NH_2_-terminal truncated fragments of tau protein -including our peptide [21, 27, 28]- is mainly present in CSF from human AD patients and secreted from cryopreserved synaptosomes following depolarizing stimulus [9, 28]. Importantly, in support of a recent report elucidating the beneficial role sustained by the immunodepletion of N-terminal projection of tau (residues 6-18) in improving memory deficits in 3XTgAD mice [20], these results hopefully prospect that passive immunization with the our newly-developed 12A12 monoclonal antibody targeting the N-terminal sequence of human protein encompassing the 26-44 aminoacidic stretch could actually represent an effective therapeutic opportunity for AD and other tauopathies. In addition, the observation that the primary and earlier target of action of pathogenic extracellular NH_2_htau appears to be the functional release of neurotransmitter from nerve terminal ends -which is followed by delayed mechanisms of cell degeneration- are in agreement with previous observations reporting that synaptic impairment precedes the tardive cell loss in patients and in experimental models of tauopathies, including AD [114]. Finally, the findings that pathogenetic NH_2_-truncated tau is capable of negatively affecting the presynaptic glutamate release from isolated synaptosomes in Ca^2+^ -dependent manner are also in line with previous electrophysiological, behavioral, biochemical and morphological evidence showing that mutated htau -in addition to its classical postsynaptic actions [17] and prior to frank neuronal loss- is also able to induce an early synaptic dysfunction by means of presynaptic mechanisms including changes in Ca^2+^ homeostasis, altered probability of neurotransmitter release, ultrastructural abnormalities and reduced expression of presynaptic markers [115, 116, 117, 118, 119, 120, 121].

### Extracellular NH_2_-truncated tau disrupts Ca^2+^- dependent glutamate release at presynaptic terminals: potential pathomechanism(s) and relevance to neurodegeneration

A causal association between intracellular accumulation of pathological tau species and dysregulation of calcium homeostasis in the brains of AD patients and in cellular and animal models has been already described [55, 56]. As shown by functional analyses carried out on purified synaptosomal preparations (Figures 3-4), in the present study we report that the secreted and pathologically AD-relevant NH_2_htau fragment from the extracellular milieu limits the depolarization-evoked glutamate release in a potent (at nanomolar concentrations) and rapid manner (within few minutes), likely acting on regulation of intracellular Ca^2+^ dynamics at the level of stimulus-exocytosis cascade. To this point, although several mechanisms should be taken into account, we favor the hypothesis of an indirect effect on intrinsic viscosity of lipid bilayers of neuronal plasma membrane leading to a plausible partitioning/diffusion of the (Cav)2.1 and (Cav)2.2 voltage-gated calcium channels (VDDC, P/Qtype and N-type respectively) which are known to be located into lipid-rafts and responsible for coupling of neuronal depolarization with neurotransmitter release at the level of presynaptic terminals [122]. In this regard, pathological tau has also been reported to accumulate within these cell-surface detergent-resistant microdomains of the plasma membrane in aging Tg2576 transgenic mice and AD brains [123] and the N-terminal projection domain of tau protein associates to lipid-rich rafts of plasma membrane in a phosphorylation-dependent manner [41]. Besides, our biochemical and biophysical results from kinetic studies of evoked Ca^2+^transient along with Western blotting and DSC analyses (Figures 2-3-4-5) further support the above observations demonstrating that: (i) the dose-dependent impairment of glutamate exocytosis induced by NH_2_htau attached to surface of isolated nerve endings occurs upstream of Ca^2+^ dynamics out of the action mechanism of neurotransmitter release induced by external high [KCl] which is known to involve only the depolarization-evoked activation of voltage-dependent Ca^2+^ channels [57, 59] and not of other ion-channels modulating the synaptic terminal excitability; (ii) treatment of neuronal cultures with NH_2_htau does not induce *per se* up to 48h any significant alteration in the protein expression level of some components of cytoskeleton and/or of release machinery which is relevantly involved in controlling the excitatory neurotransmission, excluding the possibility of a direct effect downstream of Ca^2+^ entry on synaptic vesicle trafficking/ exocytosis apparatus and on axonal transport; (iii) upon its prompt interaction with the surface of biomembrane-mimicking environments, the external application of NH_2_htau gives rise to a profound phase segregation of lipid bilayer leading to a selective enrichment in more rigid, raft-like microdomains which are largely accepted to be the cellular sites where functional assembly of SNARE (soluble N-ethyl-maleimide-sensitive fusion protein attachment protein receptor) components of vesicular exocytotic machinery with presynaptic P/Q calcium channels occurs [122]. In addition, our preliminary data (not shown) from microscopy visualization of membrane fluidity by means of 2-Dimethylamino-6-lauroylnaphthalene (Laurdan) fluorescence points to a significant perturbation in lipids packing upon short-term exposure of neuronal cultures to NH_2_htau but not to its reverse control sequence. Interestingly, it has been reported that α-synuclein associates with lipid-rafts microdomains [52] in a similar way of N-terminal end of tau [16] and that, when extracellular-applied in its monomeric form, is able to early perturb calcium homeostasis by affecting the portioning of several cell surface-associated proteins [124, 125]. Therefore consistent with our immunofluorescence stainings (Figure 2), it would be reasonable to hypothesize that secreted NH_2_htau might change the membrane fluidity/composition upon its prompt interaction with presynaptic boutons and alter thus the distribution and/or insertion of Ca^2+^ channels into sorting /signaling lipid platforms as well as the association of these channels with appropriate effector proteins, thus inhibiting their activities [126]. Of note, biophysical parameters (bending, fluidity and thickness) underlying the reciprocal spatial organization between lipids membranes and surface-associated proteins have been largely accepted to actively participate in presynaptic functions and to drive the neurotransmitter release in neurons via regulation of proteins trafficking and activity. In line with our DSC analyses, previous studies have also proved that one of the initial steps in neurotoxic biological effects evoked by rather low concentrations of Aβ stems from a “membrane-disordering effect” caused by alteration in acylchain layer and, thus, in fluidity of cell membranes [127]. Additional studies are required to test this possibility and further experiments are under current investigation to better address the presynaptic pathway(s) underlying the membrane-mediated actions of NH_2_htau in neurons. In relation to the presynaptic Ca^2+^-dependent coupling between stimulus and neurotransmitter secretion of interest is the fact that, as shown in our correlational studies of Ca^2+^ imaging and radioactivity-based measures of [3H]D-aspartate release, the peak of K^+^-evoked elevation in Ca^2+^ current turns out to be somewhat higher and to decline to a stable plateau more quickly in hippocampal isolated nerve terminals exposed to NH_2_htau than in other two experimental group (reverse- and untreated controls), resulting in overall decrease of glutamate release (compare Figure 3 to Figure 4). To this point, it’s worth stressing that the strength of glutamatergic synapses depends on both the peak and the kinetic of [Ca^2+^] transient (duration) near its sensors. It has been largely accepted that depolarization of synaptosomes by elevated KCl initiates a rapid “spike” of Ca^2+^ current within 1-2 s of stimulation followed by a more extensive slow plateau. The K^+^ -evoked secretion of glutamate neurotransmitter from synaptic terminals does not correlate with the increase in [Ca^2+^] bulk but occurs for the large part during its late recover to a plateau, undergoing a biphasic release which may reflect a dual localization of releasable vesicles at the active zone and in the cytoplasm [128]. Therefore, we argue that greater but shorter signal of activity-dependent Ca^2+^ transient could account for pathophysiological deficits induced by extracellular NH_2_htau on neurotransmission at the presynaptic level (Figure 3j-3k). Furthermore our findings on acute and potent inhibitory action exerted by pathogenetic NH_2_-truncated tau on glutamate release from isolated synaptosomes (Figure 4i) are also in line with the emerging concept that the unbalance in network activity occurring *in vivo* in prodromal AD subjects is more likely to reflect a decreased capacity of cerebral neurons to cope with existing glutamate level that becomes toxic at concentration that normally shows no harmful effect. Relevantly, magnetic resonance spectroscopy has revealed a significantly lower level of glutamate in AD brains compared to healthy controls and patients with mild cognitive impairment [129]. In line, soluble pathological tau species have been recently proved to be able to change the neuronal electrical properties in the rTg4510 (mutation P301L) mouse model of tauopathy, provoking a diminution in global neuronal activity with slower spontaneous oscillations and a reduced firing rates, prior to significant cell death or synapse loss [130].

### Pathological molecular/structural determinants at N-terminal extremity of human tau protein: novel opportunities for tau-based immunotherapy in AD and other tauopathies

In addition to aberrant calcium homeostasis and imbalance in neurotransmitter release, selective and regional loss of presynaptic terminals, neurite retraction, breakdown of microtubules, prominent loss of mitochondria which specifically reside at nerve endings are also discernable in transgenic animal model of tauopathy and in AD patients by sensitizing neurons to “dying back” prior to overt cell death [30, 80, 81, 131, 132, 133]. Consistently, our *in vitro* biochemical data from Western blotting and immunofluorescence analyses (Figure 5-6-Supplementary Figure 4) show a dose-dependent diminution in selective markers involved in trafficking/priming of presynaptic vesicles along with a pronounced degeneration of neuronal processes and reduction in mitochondrial density only after 48h incubation time with low doses of NH_2_htau, in absence of any significant global change in cell viability (Supplementary Figure 3) which conversely appeared evident later on (72-96h). These findings are relevant because synaptotoxicity, axonopathy and mitochondrial abnormalities induced *in vitro* by chronic and sublethal exposure of mature hippocampal primary neurons to NH_2_htau recapitulate a few important *in vivo* biochemical and morphological changes that closely resemble the pre-symptomatic neuropathologic stages of human tauopathies. To the point and in line with our *in vivo* previous results [26], recent findings have endorsed a crucial role of tau dysfunction in affecting biology and density of mitochondria located at terminal ends (i.e. synaptic mitochondria) [89]. Proteomic analyses have also shown that presynaptic compartment, along with a significant impairment in dynamic stability of microtubules and reduction in number of synaptic vesicles, are preferentially deregulated *in vivo* into transgenic mice expressing another toxic human truncated tau fragment (aa 151-391) [134]. In addition, the A152T tau mutation -a tauopathies risk factor which lies within the N-terminal projection region of protein not involved in interaction with microtubules - has been recently shown to perturb presynaptic neurotransmission and mitochondrial distribution in C.elegans worms, in absence of any accumulation of insoluble intracellular aggregates [135]. Therefore and in a similar way of extracellular-added monomeric Aβ 1-42 whose continuous exposure (i.e. without replacing the culture media up to 72-96h) to cortical primary neurons is required to achieve maximal neurotoxicity due to its sequential binding to membranes, a threshold level of membrane-bound NH_2_htau could be needed to trigger a full-blown neuronal cell death. Alternatively, neurotoxicity initiated by binding of extracellular-added monomeric NH_2_htau to membranes could depend on the activation of long-term intracellular signaling transduction pathways which affect one or more second messengers controlling, directly or indirectly, the neuronal bioenergetics, cytoarchitecture and synapses integrity. Finally and in agreement with our DSC kinetic analyses (Figure 2) displaying that NH_2_htau is able to interact with the deep hydrocarbon region of lipid bilayer only after longer incubation times (48-72h), it’s also hypothesizable that -following its initial adsorption on the external side of plasma membrane- a residual amount of this peptide could be later internalized and even undergo an intracellular misfolding which, in turn, causes the delayed loss of synapses, neurite retraction and mitochondrial deficit. Consistent with these findings, monomeric exogenous tau has been demonstrated to be up-taken by neurons [136, 137] and aggregate into endosomes [136]. Interestingly, high intracellular levels of pathogenetic NH_2_htau are able to interfere with the mitochondrial biology leading to drop of energy (ATP) and eventually to neuronal death, as we previously reported [138]. Furthermore, quantitative mass spectrometry following co-immunoprecipitation with an antibody just encompassing the extreme N-terminal domain (27-40aa) of tau protein have also revealed that numerous pre-synaptic proteins involved in vesicle docking/fusion -together with a large number of mitochondrial markers- are selectively pulled-down from mice brains [139] .

Concerning a direct role of self-aggregation in affecting synaptic function, it’s also worth mentioning that the oligomerization process of full length or truncated tau species is not essential for internalization, secretion and propagation [13, 22, 62, 136, 140] and that truncation can cause degeneration independently of its aggregation [110]. Our observations from CD spectra, ThT-binding fluorescence intensity measurements and SAXS analysis confirm the unstructured/flexible and monodisperse nature of NH_2_htau in water environment under current experimental conditions along with EOM study unraveling its intrinsic propensity to adopt specific conformational ensembles which might account for its potent *in vitro* biological effects (Figures 1,7,8). Therefore, although we cannot completely rule out that NH_2_htau can undergo temporary structural changes [35] as well as membrane-induced conformational transitions [42, 43] facilitating its folding/clustering in amorphous and/or partially structured aggregates over longer periods of times, our findings clearly demonstrate that this N-terminal tau peptide adopts a pathological conformation being certainly *in vitro* toxic when extracellular-added to neurons in its prevailing monomeric form.

It’s worth pointing out that , in the present work we used concentrations for NH_2_htau ranging from 1nM to 1µM which are within the physiological range of unbound/free tau [61, 65] and within the range previously used by other authors in assessing the extracellular role of full length tau (62) or its C-terminal [141] and N-terminal [13] active fragments on cell lines and primary neurons respectively. Furthermore, it’s noteworthy that: (i) the activity-dependent secretion of full length tau may occur through synaptic transmission [142] and (ii) the pre-synaptic release of longer 20-22kDa tau fragment occurs following K^+^ depolarization of AD terminal ends [28]. Therefore, although the amount of extracellular NH_2_htau required to affect synaptic functions is higher than the diffusive levels of tau found in AD patient CSF or in the conditioned media from neuronal cultures and in interstitial fluids, it’s more likely to assume that the secretion of soluble tau species through a synaptic mechanism would necessarily generate local concentrations at the release site, and likely site of action, which are significantly higher than its circulating levels [13].

Regarding the observation that no significant effect on basal synaptic exocytosis was found out in neurons upon treatment with subtoxic doses of extracellular NH_2_htau, compelling evidence has proved that the stimuls-independent discharge at the level of the release machinery and the action potential-triggered neurotransmitter release: (i) rely on independent neuronal signal transduction pathways and on divergent presynaptic machineries and/or postsynaptic targets that may even operate in a spatially segregated manner; (ii) are not linked with respect to their relative activities because modulations of spontaneous release are not always accompanied by corresponding changes in evoked exocytosis. Therefore, following binding to plasma membrane and consequent alteration in the lipid bilayer fluidity as we detected by DSC (Figure 2), it’s conceivable that extracellular NH_2_htau might perturbate the localization and, then, the activation of one or more membrane-associated proteins which are selectivity associated to regulated neurosecretion. The specific effect induced by extracellular NH_2_htau on the movement/distribution of distinct membrane-associated proteins in lipid rafts and, then, on the differential regulation of evoked versus spontaneous neurotransmitter release, is possible to be dependent on the different strength of association of proteins with membrane bilayer, or on the differential role of lipid raft elements in the maintenance of proteins cluster and in the confinement of distinct proteins in their specific site of localization [125].

Finally it’s also noteworthy that, although the extracellular NH_2_tau 26-44 fragment (i.e.NH_2_htau) only comprises 19 amino acid sequence of over 400 amino acids included in full length tau sequence, it represents the minimal biologically active moiety of longer 20-22kDa parental peptide [27, 33, 34]. Therefore, given that (i) molecular characterization of identity of the extracellular toxic tau species is mandatory to design a best-targeted and more effective immunotherapy relying on the specific, epitope-directed and antibody-mediated depletion [4, 5]; (ii) the NH_2_ 26-44aa is critical aminoacidic stretch representing the biologically active moiety of 20-22kDa AD-relevant secreted NH_2_-truncated tau forms [24, 27, 34], our findings can be particularly relevant for tau physiopathology in the field of AD and other tauopathies, helping to design more beneficial tau-directed and disease-modifying *in vivo* curative approaches. To this regard, it’s worth noting that passive immunotherapy with HJ 8.5 targeting the N-terminal projection domain of human tau (residues 25-30) has proved to be succesful in inhibiting the transcellular propagation of tau and improving cognitive deficits in P301S tau transgenic mice [143]. In addition, based on the encouraging *in vivo* results from tauopathy animal models [20, 143, 144], clinical trials with a set of monoclonal tau antibodies binding to and clearing the extracellular and/or intracellular pathological species including the extreme N-terminal region of human protein are currently in progress [5].

### CONCLUDING REMARKS

In summary, the present investigation not only confirms and extends the notion that extracellular tau is *per se* harmful for neurons [13, 23, 62] but also opens novel and potentially more effective therapeutic opportunities aimed at preventing the early impairment in synaptic plasticity and memory caused by one of the actually secreted [21, 27, 28] and, consequently, pathologically relevant N-terminal truncated species in human tauopathies.

## MATERIALS AND METHODS

### Chemicals and antibodies

Brain Total Lipid Extract (TLBE), 1, 2-dimyristoyl-sn-glycero-3-phosphocholine (DMPC) and 1, 2-dimyristoyl-sn-glycero-3-phospho-L-serine (DMPS) 1, 2-palmitoyl-oleoyl-sn-glycero-3-phosphocholine (POPC) and 1, 2-palmitoyl-oleoyl-sn-glycero-3-phosphoserine (POPS) were purchased from Avanti Polar Inc. (Alabaster, AL). All N-fluorenylmethoxycarbonyl (Fmoc)-protected amino acids, Fmoc-NH-(PEG)11-COOH, 2-(1-H-benzotriazole-1-yl)-1, 1, 3, 3-tetramethyluronium tatrafluoroborate (TBTU), and NovaSyn TGR resin were obtained from Novabiochem (Switzerland); N, N-diisopropyl-ethylamine (DIEA), N-hydroxybenzotriazole (HOBt), DMF (peptide-synthesis-grade), triisopropylsilane (TIS), trifluoroacetic acid (TFA), Thioflavin T (ThT), Sodium dodecyl sulfate (SDS), 2, 2, 2-Trifluoroethanol (TFE), N, N-diisopropylethylamine (DIEA), N, N-dimethylformamide, piperidine, triisopropylsilane (TIS), trifluoroacetic acid (TFA) and all salts used for buffer preparation were purchased from Sigma-Aldrich (St.Louis, MO) with a purity of 99%. SynaptoRed C2 (FM4-64) 70021, SynaptoGreen C4 (FM1-43) 70022 were from Biotium (Hayward, CA); Calcium Indicator (Fluo-3AM) F1242 was from Thermo Fisher Scientific (Massachusetts, MA).

The following antibodies were used: β-III tubulin antibody rabbit ab18207 Abcam; β-III tubulin antibody mouse (clone 2G10) T8578 Sigma-Aldrich; β-actin mouse S3062 Sigma-Aldrich; Synapsin I antibody rabbit AB1543P Millipore Corporation; PSD95 antibody (clone 7E3-1B8) mouse MAB1598 Millipore; PSD95antibody (clone: 6G6-1C9) mouse ADI-VAM-PS002 EnzoLife Science; synaptophysin antibody mouse sc-17750 Santa Cruz; Calnexin antibody ( clone C-20) goat sc-6465 Santa Cruz ; MAP-2 antibody mouse MAB3418 Millipore; dynamin antibody mouse BD Transduction Laboraories 610246; GAPDH antibody (clone GAPDH-71.1) G8795Sigma-Aldrich ; SNAP25 antibody mouse (clone SMI81) Biolegend 836301; SV2A antibody (clone E-8) mouse sc-376234 Santa Cruz; α-synuclein antibody (clone 42) mouse 610786 BD Transduction Laboratories; α-synuclein antibody rabbit S3062 Sigma-Aldrich; synaptotagmin antibody (clone ASV48) mouse SYA-148 Stressgen; anti complex IV subunit I MTCO1 (clone 1D6E1A8) ab14705 MitoSciences; NMDAζ1 antibody (C-20) goat sc-1467 Santa Cruz; vGLUT1antibody rabbit 135 302 Synaptic System ; Anti- caspase 3cleaved (active) form antibody rabbit AB3623 Millipore; golgin 97 (E-16) antibody goat sc-74632 Santa Cruz.

### Synthesis, purification, labeling and preparation of tau peptides

NH_2_ htau 26-44 and its reverse sequence control peptide were synthesized and purified using reverse-phase HPLC (D.B.A., Milan, Italy). Peptides mass and purity (>99%) were confirmed by reverse-phase HPLC and electrospray/ion trap mass spectrometry. A lyophilized powder of peptides was dissolved in at a stock concentration of 1 mM by brief stirring. Aliquots were stored at -80°C prior to use. The working solution was diluted with PBS or ultrapure water at a concentration of 50 μM at 4°C and used immediately. Peptide preparations were analyzed by Sypro Ruby protein staining and SDS-PAGE Western blot for each experiment.

NH_2_ htau 26-44 :NH_2_ QGGYTMHQDQEGDTD AGLK- COOH

Reverse sequence control peptide :NH_2_ KLGADTDGEQDQHM TYGGQ- COOH

FITC- conjugated NH_2_htau 26-44 was synthesized and purified (>99%) using reverse-phase HPLC (Biosynthesis, U.S.A.).

### Generation of the N-terminal tau 12A12 antibody (26-36aa)

Affinity-purified mouse monoclonal antibody directed against the extreme N-terminal 26-36 aa of human tau protein (12A12) was produced, purified and characterized by Monoclonal Antibodies Core Facility (MACF) at EMBL- Monterotondo, Rome, Italy (Dott.Alan Sawyer).

12A12 was generated by immunizing mice with a peptide of amino acids 26-36 aa of hT40 (D25(NH_2_ QGGYTMHQDQ-COOH epitopes). The specificity of this mAb (IgG isotype) was verified by Western blot analysis (Figure 1f-1g) and enzyme-linked immunosorbent assay (ELISA) test (95% sensitivity and 90% specificity).

### Preparation of artificial biomembrane model system

We used large unilamellar vesicles (LUVs) composed of TLBE or DMPC/DMPS (7/3 molar ratio). Model membranes were prepared as described elsewhere [47]. Briefly, aliquots of lipid stock solutions in chloroform were dried by using a stream of dry nitrogen gas and evaporated under high vacuum to dryness in a round-bottomed flask. To obtain multilamellar vesicles (MLVs), the resulting lipid film was hydrated with an appropriate amount of phosphate buffer (10 mM buffer, 100 mM NaCl, pH = 7.4) and dispersed by vigorous stirring in a water bath. LUVs were obtained by extruding MLVs through polycarbonate filters (pore size = 100 nm, Nuclepore, Pleasanton, CA) mounted in a mini-extruder (Avestin, Ottawa, ON, Canada) fitted with two 0.5 ml Hamilton gastight syringes (Hamilton, Reno, NV). Samples were typically subjected to 23 passes through two filters in tandem and as recommended elsewhere. An odd number of passages were performed to avoid contamination of the sample by vesicles that might not have passed through the filter.

### Circular dichroism

The CD spectra were obtained at 25°C (if not differently specified) under a constant flow of N2 on a Jasco J-810 spectropolarimeter equipped with a Peltier thermoelectric type temperature control system. Experimental measurements were conducted in several condition, using 1 cm path length cuvettes. The CD measurements were carried out under a variety of experimental conditions, including different pH levels and membrane-mimicking enviroments (i.e negatively charged SDS, anionic-zwitterionic POPC/POPS LUV and water/TFE mixtures). The CD spectra were recorded in the UV region (190-260 nm) with peptide concentration of 10 µM. CD intensities are expressed as molecular ellipticity.

### ThT assay

The kinetics of amyloid fiber formation were measured using the increase of fluorescence emission upon binding of commonly used amyloid specific dye, thioflavine T (ThT). Samples were prepared by adding 5 µL stock solution of tau26-44 or tau44-26 to 100 µL of 10 mM phosphate buffer solution pH 7.4, 100 mM NaCl or to 100 µL of a TLBE 7/3 LUVs solution, containing 60 µM ThT (final peptides concentration was 20.0 µM). Experiments were carried out in Corning 96 well non binding surface plates. Time traces were recorded using a Varioskan plate reader (λecc 440 nm, λem 482 nm) at 37 °C, shaking samples for 10 seconds before each read. Time traces are the average of three measurements.

### Differential scanning calorimentry (DSC)

The interaction of NH_2_htau and reverse control peptide with model membranes was tested by performing DSC experiments. Samples were prepared by adding the proper amount of a stock solution (800 µM) of both tau peptides to 1 ml of LUV DMPC/DMPS 7/3 200 µM. The final peptides concentration was 20 µM. Five independent samples were prepared in order to perform experiments every 24 hours up to 72 hours . All the experiments were collected from 10 to 40 °C, 1 °C/min scanning rate at the pressure of 3 atm using a nanoDSC (TA instrument).

### Animals

All protocols involving animals were performed in accordance with the guidelines established by the European Communities Council (Directive 2010/63/EU of 22 September 2010). Experiments involving animals were performed in accordance with the relevant approved guidelines and regulations accepted by the Italian Ministry of Health and approved by the Ethical Committee on animal experiments of EBRI “Rita Levi-Montalcini” Foundation (Rome, Italy).

### Synaptosomes preparation

Mouse hippocampal purified synaptosomes were prepared by homogenizing tissue in 10 volumes of 0.32 M sucrose, buffered to pH 7.4 with Tris-(hydroxymethyl)-amino methane [Tris, final concentration (f.c.) 0.01 M] [75]. The homogenate was centrifuged at 1, 000 x g for 5 min and the supernatant was stratified on a discontinuous Percoll gradient (2%, 6%, 10% and 20% v/v in Tris-buffered sucrose) and centrifuged at 33, 500 x g for 5 min. The layer between 10% and 20% Percoll (synaptosomal fraction) was collected and washed by centrifugation. In a set of experiments, the tissue was homogenized in buffered sucrose containing 1 mM 1, 2-bis-(2-aminophenoxy) ethane-N, N, N’, N’, tetra-acetic acid (BAPTA), in order to entrap this agent into subsequently isolated synaptosomes [75]. The synaptosomal pellets were resuspended in a physiological solution with the following composition (mM): NaCl, 140; KCl, 3; MgSO_4_, 1.2; CaCl_2_, 1.2; NaH_2_PO_4_, 1.2; NaHCO_3_, 5; HEPES, 10; glucose, 10; pH 7.2-7.4. To ascertain whether fractionated preparations were really enriched in synaptic terminals and free of any contaminations from neuronal perikarya, Western blotting analysis was carried out to check the purity of samples by probing with antibodies against the presynaptic protein synapthophisin and cytosolic GAPDH, as previously reported [27].

### Synaptosomal glutamate release

Synaptosomal glutamate release was estimated as previously reported [74, 75, 77] . In details, isolated hippocampal ending nerves were incubated for 45 min at 37°C in a rotary water bath in the presence of [3H]D-aspartate ([3H]D-ASP; a non-metabolizable analogue of glutamate used to label the synaptosomal glutamate-releasing pools used at final concentration 50 nM). Aliquots of synaptosomal suspension (5-10 μg protein) were equally distributed on microporous filters placed at the bottom of a set of parallel superfusion chambers maintained at 37 °C (Superfusion System, Ugo Basile, Comerio, Varese, Italy) [74]. Superfusion was then started with standard medium at a rate of 0.5 ml/min and continued for 48 min. After 39 min of superfusion to equilibrate the system, synaptosomes were transiently (90s) exposed to high K^+^ containing medium (12 mM, NaCl substituting for an equimolar concentration of KCl), and then fractions were collected according to the following scheme: two 3-min fractions (basal release), one before (t = 36-39 min) and one after (t = 45-48 min), a 6-min fraction (t = 39-45 min; evoked release). When indicated, synaptosomes were superfused with medium in which Ca^2+^ ions were omitted to prevent any Ca^2+^-dependent releasing activity.Collected fractions and superfused synaptosomes were measured for radioactivity. The amount of radioactivity released into each superfusate fraction was expressed as percentage of the total radioactivity. The K^+^-induced overflow was estimated by subtracting the neurotransmitter content the first and the third fractions collected (basal release, b1 and b3) from that in the 6-min fraction collected during and after the depolarization pulse (evoked release, b2). In a set of control experiments performed to evaluate the Ca^2+^-dependency of the induced [3H]d-ASP release, the superfusion medium was replaced, starting from t = 20 min, with a medium containing 0.1 mM Ca^2+^ and 500 μM EGTA. In some experiments, synaptosomes were incubated 30 min (15 min before and during [3H]d-Asp labelling) in the presence of 100 μM 1, 2-bis-(2-aminophenoxy)-ethane-N, N, N′, N′-tetraacetic acid, tetraacetoxymethyl ester (BAPTA-AM).

### Vesicles exocytosis FM1-43 or FM4-64 assay

Depolarization-dependent FM1-43 or FM4-64 destaining was used to measure synaptic vesicle fusion events with the plasma membrane on intact nerve terminals (i.e., synaptosomes) [59, 71, 72, 73]. Isolated synaptosomal fractions from mature rat primary hippocampal neurons (15DIV) seeded on coverslips were incubated in physiological salt solution (PSS; NaCl 140 mM, glucose 11.5 mM, KCl 5.9 mM, MgCl_2_ 1.4 mM, NaH_2_PO_4_ 1.2 mM, NaHCO_3_ 5 mM, CaCl_2_ 1.8 mM, HEPES 10 mM) and then with FM1-43 (50 μM) for 2 min, followed by application of high KCl 30 mM to intrasynaptically load the fluorescent dye. After 1 min, synaptic preparations were washed twice to remove non-internalized FM1-43 and placed on an incubation chamber of a time-lapse system composed by an inverted fluorescence microscope (TiE; Nikon, Japan), equipped with an incubation chamber (Okolab), a cooled CCDcamera (Clara;Andor), a Perfect Focus System to avoid z-axis focus fluctuations and a Niss Elements imaging software (Nikon). After 1 min a baseline was established, synaptoneurosomes were incubated (t = 1) with either NH_2_htau peptide or its reverse sequence control (both 1µM) for additional 5 min and then (t = 6) stimulated by application of 30mM KCl which we previously stated in pilot experiments to be the minimum concentration able to induce robust responses in our samples. Video recordings were performed with a 40×oil objective (N.A 1.4) for at least 10 min and 14 bit images were captured by 200 ms exposure time every 2 s at room temperature (25°C). After fluorescence recordings, one differential interference contrast (DIC;Nomarski) image of the field was taken for visual inspection of global morphological features (data not shown). Video images were analyzed by using the Spot module of the ImarisSuite 7.6® software (Bitplane A.G., Zurich, Switzerland) by semiautomated application of 1.5 μm radius diameter mask on fluorescent puncta. The criteria for fluorescence puncta inclusion in the data analysis were the spherical shape ranging between 0.5-1.5 micron and the stimulation-dependent destaining response. Destaining time courses were generated by normalization of each fluorescence spot trace by using the formula F _i_/F_0_ where F_0_ is the fluorescence before stimulus addition (average of 10 time points of the image t-stack) and F_i_ is the fluorescence at each time point. Quantification of FM1-43 exocytosis responses was accomplished by calculating the average percentage of fluorescence loss and a group of control experiments was also contextually run to test the specificity of dye loading and destaining, as following: (i) incubation in Ca^2+^-free Ethylene Glycol TetraAcetic acid (EGTA)-containing medium drastically reduced both the FM1-43 loading and inhibited the K^+^ stimulus-induced dye release; (ii) the FM1-43 photobleaching rate was measured in absence of the K^+^ stimulus. To verify whether NH_2_htau could induce *per se* synaptic release, we additionally performed a few control experiments using the same experimental setting conditions with the exception that the NH_2_htau was incubated with the synaptosomes and the KCl stimulus was substituted by saline addition.

### Intracellular calcium imaging in cultured neurons and isolated synaptosomes

Relative changes in the cytosolic Ca^2+^ concentration were measured using the Ca^2+^ -sensitive fluorescent dye indicator Fluo-3 AM. Hippocampal primary neurons and isolated hippocampal ending nerves were incubated for 45 min in the dark at room temperature with 2 µM Fluo-3 AM dissolved in 0.02 % pluronic acid (both from Biotium, Hayward, CA) in physiological salt solution (PSS; NaCl 140 mM, glucose 11.5 mM, KCl 5.9 mM, MgCl_2_ 1.4 mM, NaH_2_PO_4_ 1.2 mM, NaHCO_3_ 5 mM, CaCl_2_ 1.8 mM, HEPES 10 mM). To allow for completion of Fluo-3 AM de-esterification process, cultures were washed and incubated in PSS alone for 10-15 min at 37 °C and then visualized and recorded by using a time-lapse system mounted on an inverted fluorescence microscope (TiE; Nikon, Japan), equipped with: (i) an incubation chamber (Okolab, Italy), (ii) a cooled CCD camera (Clara; Andor), (iii) filters for Differential Interference Contrast (DIC; Nomarski) acquisitions, (iv) Perfect Focus System to avoid z-axis focus fluctuations and a (v) Niss Elements imaging software (Nikon). Video recordings were performed with a 20X objective, for at least 20 min, and 12 bit images were captured by 200 ms exposure time every 2 s at room temperature (25°C). After 1 min a baseline was established, neurons were incubated (t = 1) with either NH_2_htau peptide or its reverse sequence control (1µM) for additional 5 min and then (t = 6) stimulated by 30 mM KCl addition, which we previously verified in pilot experiments to be the minimum concentration able to induce robust responses on our *in vitro* cultures. After fluorescence recordings, one DIC image of the fluorescence field was taken for visual inspection of morphological features. Fluorescence intensity changes were calculated as (F-F_0_)/F_0_, where F was the fluorescence intensity measured in each frame after stimulation and F_0_ the basal fluorescence level calculated by averaging the intensity values 20 sec before the peak. Video images and pixel intensities were evaluated afterwards, by using Imaris Suite 7.6® Track module (Bitplane A.G., Zurich, Switzerland) and Image J 1.4 (imagej.nih.gov/ij/) softwares by applying a 1.5 μm radius diameter mask on fluorescent puncta. However, some puncta which visually displayed a faint Fluo-3 staining were not detected by the Track module, owing to a low responses to KCl stimulation, and therefore were not included in the analysis. Inclusion criteria for data analysis were: (i) puncta labeling with Fluo-3, (ii) puncta showing a prompt Ca^2+^ rise and sustained Ca^2+^ response (which was more than 20% increase over resting baseline), (iii) puncta returning to fluorescence baseline level. For all calcium signals, amplitude, time to peak, peak to baseline and overall duration of the response were determined. In few control experiments performed to evaluate the source of Ca^2+^ increase, a medium containing 0.1 mM Ca^2+^ and 500 μM EGTA was used and no statistically significant fluorescence variation and/or typical curve shape was detected.

### Neuronal culture and treatments

Hippocampal neurons were prepared from embryonic day 17-18 (E17/E18) embryos from timed pregnant Wistar rats (Charles River), as we previously reported [34]. In detail, the hippocampus was dissected out in Hanks’ balanced salt solution buffered with HEPES and dissociated via trypsin/EDTA treatment. Cells were plated at 1 x 106 cells on 3.5-cm dishes pre-coated with poly-D-lysine. After 2 days of culturing in neurobasal medium with B-27 supplement and glutamax, cytosine arabinofuranoside was added to reduce glial proliferation. Half of the medium was changed every 3-4 days and mature neuronal cultures were infected at 15 days in vitro, as reported in [34].

### Assessment of neuronal viability

Cell viability was quantified by counting the number of intact nuclei, after lysing in detergent-containing solution and by the MTT tetrazolium salt assay, as reported in [26, 33, 34].

### Immunofluorescence

Following treatment, neuronal cultures were washed twice with PBS and fixed in 4% (w/v) paraformaldehyde for 15 min at room temperature. Cells were permeabilized with 0.1% (v/v) Triton X-100/PBS pH 7.4 for 4 min at room temperature. Coverslips were saturated with 2% BSA and 10% normal goat serum (NGS) for 3h followed by incubation overnight at 4 °C in a humidified chamber with primary antibodies. Unbound antibody was removed by three washes and bound antibody was detected by incubation with donkey anti-rabbit Alexa 488 (1:300) and donkey anti-mouse rhodamine- conjugated (1:300) secondary antibodies (Invitrogen) at room temperature for 30 min. Nuclei were stained with nuclear marker 4, 6-diamidino-2-phenylindole dihydrochloride (DAPI; Sigma, St. Louis, MO, U.S.A.) 1:1000 in PBS for 5 min. Controls were performed by omitting either the primary or the secondary antibody. Images are representative of at least three independent experiments.

### Live-fluorescence protocol

Morphological detection of cell-surface NH_2_htau was performed according to procedure aimed to detect direct interaction of extracellular-added Aβ 1-42 peptide to neuronal membrane lipids rafts with several modifications [145]. In brief, live neuronal cultures were washed twice with chilled PBS 1X , incubated for 10’ with 1µM FITC-conjugated NH_2_htau (Biosynthesis, USA) at 4°C and then labeled for lipid rafts by means of TRITC- conjugated cholera toxin subunit B (CT-B), according to manual instructions (Vybrant lipid raft labeling kit, Molecular Probes). After three washes with chilled PBS 1X, cells were fixed in 4% (w/v) paraformaldehyde for 15’ min at 4°C and coverslip slides were then mounted in medium containing the nuclear counterstain, DAPI. For morphological detection of synaptosomes-surface NH_2_htau, 1µM FITC-conjugated NH_2_htau was added 5 min to synaptosomal preparations and after two washes the FM4-64 assay(see above) was performed (50µM 1’+30mM KCl 90sec). Images are representative of at least three independent experiments.

### Subcellular fractionation and isolation of membrane-containing fraction

Biochemical detection of cell-surface NH_2_htau was performed according to procedure aimed to detect direct interaction of extracellular-added Aβ 1-42 peptide to hippocampal membranes with several modifications [146]. In brief, mature hippocampal neurons were exposed to NH_2_htau or its reverse (5µM) at 4°C per 1h and then membrane-insoluble BS^3^ (Thermo Fisher) was used for crosslinking molecules on the cell surface, according to manual instructions. After that tau-treated and untreated control cultures underwent subcellular fractionation to obtain cytosol and membrane fractions. Neurons were scraped in 5mM EDTA in phosphate-buffered saline (PBS) e pelleted by centrifugation for 10’ at 2300 Xg at 4°C. Pellets (P1) were resuspended in 100µl of fractionation buffer (0.25M sucrose 1mM MgCl_2 ,_ 2mM EGTA, 25mM HEPES ph 7.4) and lysed by three cycles of freeze-thaw in liquid nitrogen. Lysated were then centrifugated at 100, 000X g for 30’ and the supernatant (cytosol fraction) was removed. The membrane-containing pellets (P2) were resuspended in 100µl of fractionation buffer and equivalent volume of loading dye. Equal amounts of proteins were analyzed by Western blotting with our 12A12 antibody directed against the extreme N-terminal 26-36 aa of human tau protein.

### Protein cellular lysates preparation

Total proteins were extracted by scraping the cells in an SDS-reducing sample buffer or lysis in ice-cold RIPA buffer (50 mM Tris-HCl pH 8, 150 mM NaCl, 1% Triton, 2 mM EDTA, 0, 1% SDS plus proteases inhibitor cocktail (Sigma Aldrich, P8340) and phosphatase inhibitor cocktail (Sigma Aldrich, P5726/P2850) and centrifuged at 4 °C for 15 min at 1000 X g. The supernatant was then collected and boiled for 5 min.

### Western blot analysis and densitometry

Equal amounts of protein were subjected to SDS-PAGE 7.5-15% linear gradient or Bis- Tris gel 4-12% (NuPage, Invitrogen). After electroblotting onto a nitrocellulose membrane (Hybond-C Amersham Biosciences, Piscataway, NJ) the filters were blocked in TBS containing 10% non-fat dried milk for 1h at room temperature or overnight at 4°C. Proteins were visualized using appropriate primary antibodies. All primary antibodies were diluted in TBS and incubated with the nitrocellulose blot overnight at 4°C. Incubation with secondary peroxidase coupled anti-mouse, anti-rabbit or anti-goat antibodies was performed by using the ECL system (Amersham, Arlington Heights, IL, U.S.A.) In a few experiments, multiple normalizations of the same filter on different loading controls, such as β-actin and GAPDH (glyceraldehyde 3-phosphate dehydrogenase), were carried. Final figures were assembled by using Adobe Photoshop 6 and Adobe Illustrator 10 and quantitative analysis of acquired images was performed by using ImageJ (http://imagej.nih.gov/ij/).

### DLS experiment and data analysis

High-purity tau peptides were suspended in PBS solution (10× PBS: 1.3 M NaCl, 0.07 M Na_2_HPO_4_ and 0.03 M NaH_2_PO_4_, pH 7.4) at the nominal concentration of 2.5 mg/ml. Subsequently, the solution was centrifuged for 10 min at 10 000g and the supernatant filtered to eliminate aggregates. DLS measurements were performed by using a Zetasizer Nano ZS (Malvern, Herrenberg, Germany) equipped with a 633-nm He-Ne laser and operating at an angle of 173°. Solvent-resistant micro cuvettes (ZEN0040, Malvern, Herrenberg, Germany) have been used for experiments with a sample volume of 40 µL.

Dynamic light-scattering (DLS) measures the intensity autocorrelation function $G_{2}\left(\tau \right)\langle I\left(t+\tau \right)I\left(\tau \right)\rangle /{\langle Ι\rangle }^{2}$, where τ is the lag time and the bracket represents the ensemble average. The G_2_(τ) can be related to the field autocorrelation function g_1_(τ) through the Siegert relation G_2_(τ) = 1+βg_1_^2^(τ), where β is an instrumental constant equal to 1 in our setup. The mathematical form of g_1_(τ) depends on the physical properties of the system investigated. For a monodisperse solution of noninteracting particles, a single exponential function with decay time τ is obtained. For a polydisperse sample, g_1_ (τ) is no longer a single exponential. In this case, the distribution of decay rates on g_1_(τ) can be taken into account by introducing a weighting function,

$$g_{1}\left(\tau \right)=\int_{0}^{\infty }P_{1}\left(r\right)e^{-\Gamma \left(r\right)\tau }dr$$ eq. 2

where P_I_ (r)dr is the intensity-weighted radius distribution function, describing the distribution of the fraction, in the interval *dr*, of the intensity scattered by a particle of hydrodynamic radius r and decay rates $\Gamma \left(r\right)=\frac{{kTq}^{2}}{6\pi \eta \;R_{H}}$ with η is the water viscosity and k the Boltzmann constant [92, 93].The value P_I_(r) can be obtained by means of a regularized Laplace inversion of the intensity autocorrelation function. In this case the intensity-weighted radius distribution is obtained by a direct numerical inversion of the DLS data. The numerical inversion is automatically performed by the Malvern instrument software.

The number-weighted radius distribution P_N_(r) can be recovered by taking into account the form factor P(qr) and the mass M, according to the following equation [93, 94],

$$PN\left(r\right)=\frac{P_{1\left(r\right)}}{P\left(qr\right)M{\left(r\right)}^{2}}$$ eq.3

The recovery of P_N_ (r) from P_I_ (r) is automatically performed by the Malvern software package. Once the P_N_ (r) and the P_I_ (r) are known, it is thus easy to determine the number-weighted average hydrodynamic radius (R_H_ )_N_:

$${\langle R_{H}\rangle }_{N}=\int_{0}^{\infty }P_{N}\left(r\right)r_{H}dr_{H^{}}$$ eq.4

### SAXS experiment and data analysis

High-purity tau peptides were dissolved in PBS solution (10× PBS: 1.3 M NaCl, 0.07 M Na_2_HPO_4_ and 0.03 M NaH_2_PO_4_, pH 7.4). Peptides concentration was carefully determined through the Guinier approximation and reported in tab. 1. Subsequently, the solution was centrifuged for 10 min at 10 000g and the supernatant was filtered to eliminate aggregates. SAXS measurements were acquired on the BioSAXS beamline (BM-29) at ESRF (Grenoble, France), equipped with a 2D detector (Pilatus 1M, Dectris). The sample to detector distance for normal operation is 2.5 m, which allows a momentum transfer of s = (4π sin θ/λ) in the range from 0.05 to 5.8 nm^−1^. A volume of 50 μL of solution was placed in a 1.8 mm diameter quartz capillary (mounted in vacuum) with a few tens of micrometer wall thickness, using an automated sample loader. The potential effect of radiation damage was evaluated performing a 10 s exposure at constant temperature without observing any radiation damage. An exposure time of 2 s at each temperature was used to avoid possible radiation damage and experimental measures were carried out at room temperature. Solvent scattering was evaluated- before and after measuring the samples - in the same capillary sample holder to allow for background scattering subtraction.

Peptides sizes were estimated by their radius of gyration R_G_ obtained with two different methods: the Guinier approximation and the Debay model. The first model states that for very small angles (s < 1/R_G_) the scattered intensity I(s) is described by the following formula:

$$I\left(s\right)=I\left(0\right)\exp\left(\frac{s^{2}{R_{g}}^{2}}{3}\right)$$ eq. 5

where I(0) is the total scattered intensity at s = 0.

Given eq. 5, the scattering intensity plotted as ln I(s) vs. s^2^ (Guinier’s Plot) is a linear function for a particle of any shape. The radius of gyration of both peptides was determined by a linear fit of the Guinier plot. However, since the presence of aggregates and oligomers in solution can lead to an overestimation of R_G_, we also used the Debye’s approximation according to which, for a Gaussian chain, the scattered intensity I(s) of the R_G_ of the chain can be expressed by the equation:

$$I\left(s\right)=\frac{I\left(0\right)}{2}\left({\left(sR_{g}\right)}^{2}-1-e^{{\left(sR_{g}\right)}^{2}}\right)$$ eq. 6

This approximation holds for a larger momentum transfer ranges with the respect to the Guinier approximation and therefore is less affected by the presence of oligomers and aggregates [95]. We investigated the peptides flexibility by means of the Kratky plot (s^2^I(s) as a function of s) that is routinely employed to qualitatively identify disordered states that are extremely frequent in the full length human tau and in almost all its fragments exception made for ^306^VQIVYK^311^ and ^275^VQIINK^281^ , two hexapeptides with a high propensity to form beta-structures [98, 99].

Given the highly flexible nature of tau and of almost its fragments, an explicit description of the structural ensemble visited by both peptides was given by means of the EOM software package [95]. Different pools of representative backbone models with different numerosities were generated for each fragment, namely 10.000, 50.000 and 100.000. The theoretical scattering intensities corresponding to these models were calculated by means of the program CRYSOL. These intensities were then used by the genetic algorithm GAJOE to select from the initial pool an ensemble of conformers providing, on the average, the best fit of the experimental SAXS data. The following GAJOE parameters were set: number of generations, 1.000; number of ensembles, 50; number of curves per ensemble, 20; number of mutations per ensemble, 10; number of crossings per generation, 20. A total of five independent pools were used for fitting each SAXS curve.

### Statistical analysis

Experiments were carried out in triplicates and repeated at least three times. Data were expressed as means ± S.D. (*n* = 4). Statistically significant differences were calculated by unpaired two-tailed t-Student’s test and one-way ANOVA followed by Bonferroni post-hoc test (*p < 0, 05; **p <0, 01; ***p <0, 0001) as indicated in the figure legends. Data from Western blot analysis and immunofluorescence studies were representative of at least three separate experiments.

## SUPPLEMENTARY MATERIALS FIGURES


