# Supplementary material for: Extracellular truncated tau causes early presynaptic dysfunction associated with Alzheimer’s disease and other tauopathies
Source: Oncotarget. 2017 Apr 22;8(39):64745–78. doi: 10.18632/oncotarget.17371 (PMC5630290; doi:10.18632/oncotarget.17371)
Supplement: Supplementary file 1 [file oncotarget-08-64745-s001.pdf]

Extracellular truncated tau causes early presynaptic dysfunction associated with Alzheimer's disease and other tauopathies

SUPPLEMENTARY MATERIALS

SAXS results

In order to obtain detailed information about the conformational ensembles visited by NH_2htau and its reverse counterpart, we analyzed the SAXS data by using the Ensemble Optimization Method (EOM). As shown in fig. 8, a significant different sampling of the phase space was observed. These results were further confirmed in five independent fits of the experimental data by using different original pools of different numerosity.

The original pools and the selected ensembles are shown in suppl. fig. 2A. The fitting procedure appears extremely stable showing that : (i) NH_2htau and its reverse counterpart are able to sample two well-defined regions of the available phase space and (ii) NH_2tau has a higher tendency to populate more extended conformations than its inverted sequence. A quantitative estimation of this tendency for both peptides is provided in the main text by the $A_{1,5}/A_1$ ratio, a parameter which is calculate by dividing the area of the peak at high R_G by that of the peak at low R_G value. The obtained $A_{1,5}/A_1$ values for each of the five different fits and the corresponding GAJOE parameters are also summarized in suppl. fig. 2B. A value of $A_{1,5}/A_1$=3.2±0.2 and of $A_{1,5}/A_1$=1.5±0.2 can be estimated for NH_2tau and its reverse counterpart, respectively.

Image analysis and quantification

Quantitative image analysis was performed by using Fiji (ImageJ 1.51; 64 bit) [147] on 5 images from two coverslips derived by three different experimental replicates for each experimental group.

Image analysis was performed under visual control to determine the threshold which subtracts background noise and takes into account neuronal structures. During image processing, the images were compared with the original raw data to make sure that no structures were introduced which were not seen in the original data series or that structures present in the original data series were not removed. To this aim, the figures for quantification have been presented with the first image as the raw image and with the second image as the thresholded or segmented (binary) image which was finally analyzed. All data presented are mean ± SD and the three groups were compared by using one-way repeated-measures ANOVA followed by Bonferroni post-hoc test.

To analyze the morphological features of the immunomarkers of Fig. 6, we used several analysis tools and we showed in the quantification of suppl. Fig.4 the same images reported in fig 6 to facilitate the experimental understanding. For βIII-Tubulin (Fig 6A, suppl. fig. 4A), we decided to analyze the neuritic thickness by using the “local thickness” plugin [148] to describe the decrease in neuritic caliber size after long-term treatment of hippocampal cultures with NH_2htau. The algorithm computes the diameter of the sphere which fits inside the neurites and is shown in the figure as rendered thickness mask in false colour ranging from 0 to 2 μm. For α-synuclein (Fig 6A, suppl. fig. 4B), the number of immunoreactive puncta was used as the index to demonstrate the loss of immunoreactivity after exposure of neuronal cultures to NH_2htau. After the images were thresholded, a filter to limit the minimum and maximum surface extension and to limit the minimum circularity (0.2) was applied and, then, the number of puncta was measured. Due to the irregular shape of the α-synuclein immunoreactive puncta, we decided to show an image displaying the puncta outlines in order to facilitate the fidelity appreciation of the analysis in respect of the raw image. For MAP2 (Fig 6B, suppl. fig. 4C) immunoreactivity, we decided to measure the neuritic mean length to show the loss of neurites following administration of NH_2htau to hippocampal cultures. To this aim we used the plugin NeuronJ [149] which is used for the tracing and quantification of elongated image structures. Tracing was applied only to neurites which could be entirely followed on the image focus plane, fragmented and discontinuous neuritis were not considered for tracing analysis as showed in the length mask image. For synaptophysin analysis (Fig 6B, suppl. fig. 4D), the same analytical approach used for α-synuclein has been applied. We decided to show puncta as a segmentation binary mask owing to the more regular shape of the synaptophysin immunoreactive puncta. For quantification of COX-I/Synapsin colocalization (Fig 6C, suppl. fig. 4E-F), with the intent of discriminating and measuring only the colocalizing (co-positive) puncta, we developed a new analysis approach. As shown in suppl. fig. 4F, we separated the channels and generated two binary masks which were then compared by using the logical Boolean operator “AND” (Process>Image Calculator>AND). This allowed us to generate an image (Boolean AND image) which contained only the puncta present in both images (co-positive puncta). To better appreciate the fidelity of our analytical approach versus the raw image, we further generated an outline mask which allowed to compare the shape and position of the co-positive puncta between the outline mask and the merge channels image.

147. Schindelin J., Arganda-Carreras I., Frise E., Kaynig V., Longair M., Pietzsch T., Preibisch S., Rueden C., Saalfeld S., Schmid B., Tinevez J.Y., White D.J., Hartenstein V., Eliceiri K., Tomancak P., Cardona A. Fiji: an open-source platform for biological-image analysis Nature methods 2012; 9(7): 676-682.

148. Dougherty R., Kunzelmann K. Computing local thickness of 3D structures with ImageJ. Microsc. Microanal. 2007; 13: 1678-1679.

149. Meijering E., Jacob M., Sarria J.C., Steiner P., Hirling H., Unser M. Design and validation of a tool for neurite tracing and analysis in fluorescence microscopy images. Cytometry 2004; Part A. 58(2):167-176.

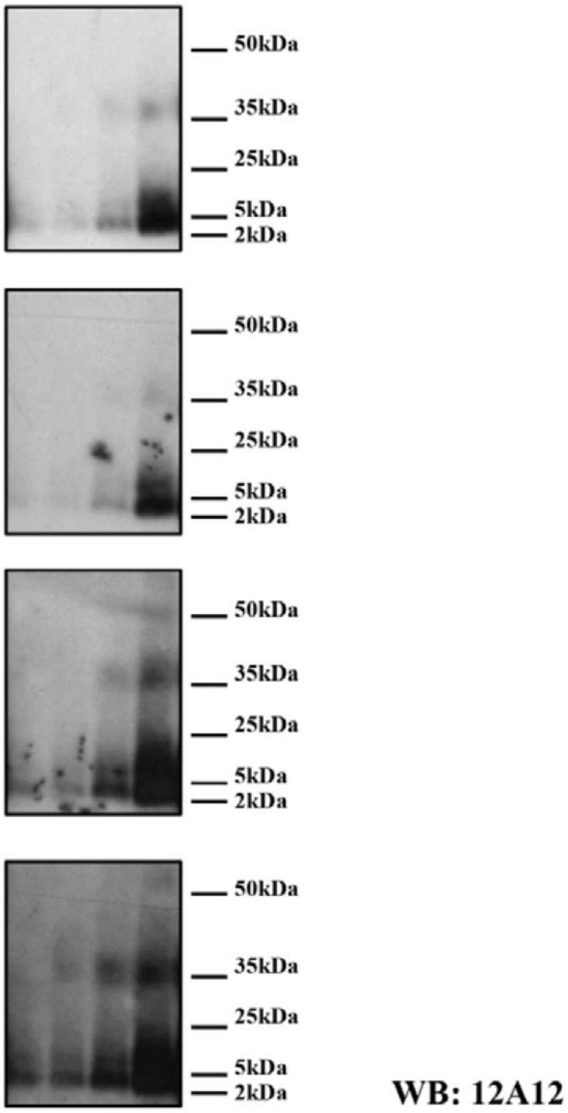

Supplementary Figure 1: Analysis of the molecular assemblies and relative distribution of monomeric and oligomeric form(s) of human NH_2tau 26-44 (i.e. NH_2htau).

Increasing amounts of NH_2htau (75-250 pmol) were analyzed by Western blotting on Bis- Tris gel 4-12% resolution gels followed by electroblotting onto a high-performing PVDF transfer membrane. The filter was probed with specific 12A12 monoclonal antibody directed against the extreme N-terminal 26-36 aa of human tau protein. Note that the overall proportion of soluble NH_2htau in oligomers turned out to be low as its monomeric concentration increased. The lowly populated fraction of higher-molecular-weight soluble oligomeric aggregates migrating around 35-50kDa was detected only after prolonged exposition of the filter, consistent with the notion that the prevailing

molecular form of NH_2htau under our standard experimental conditions appeared to be monomeric and unaggregated. Full-size WB at increasing times of image acquisition are shown.

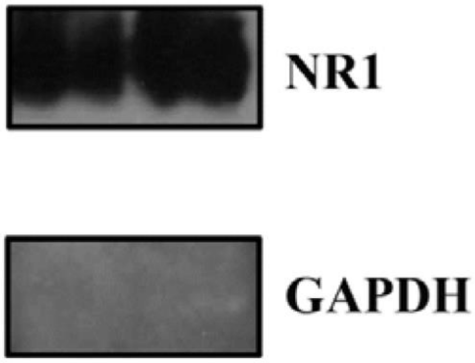

Supplementary Figure 2: Quality assessment of membranous –enriched preparations.

Membranous-enriched fractions were performed according to [161], as reported in Materials and Methods, and equal proteins amounts (70 μg) were analyzed by Western blotting with antibodies specific for the integral membrane protein N-Methyl-D-aspartate (NMDA) Receptor subunit NR1 and the cytosolic Glyceraldehyde 3-phosphate dehydrogenase GAPDH. Note that sample preparation was free of contaminants from other, non-membranous fractions.

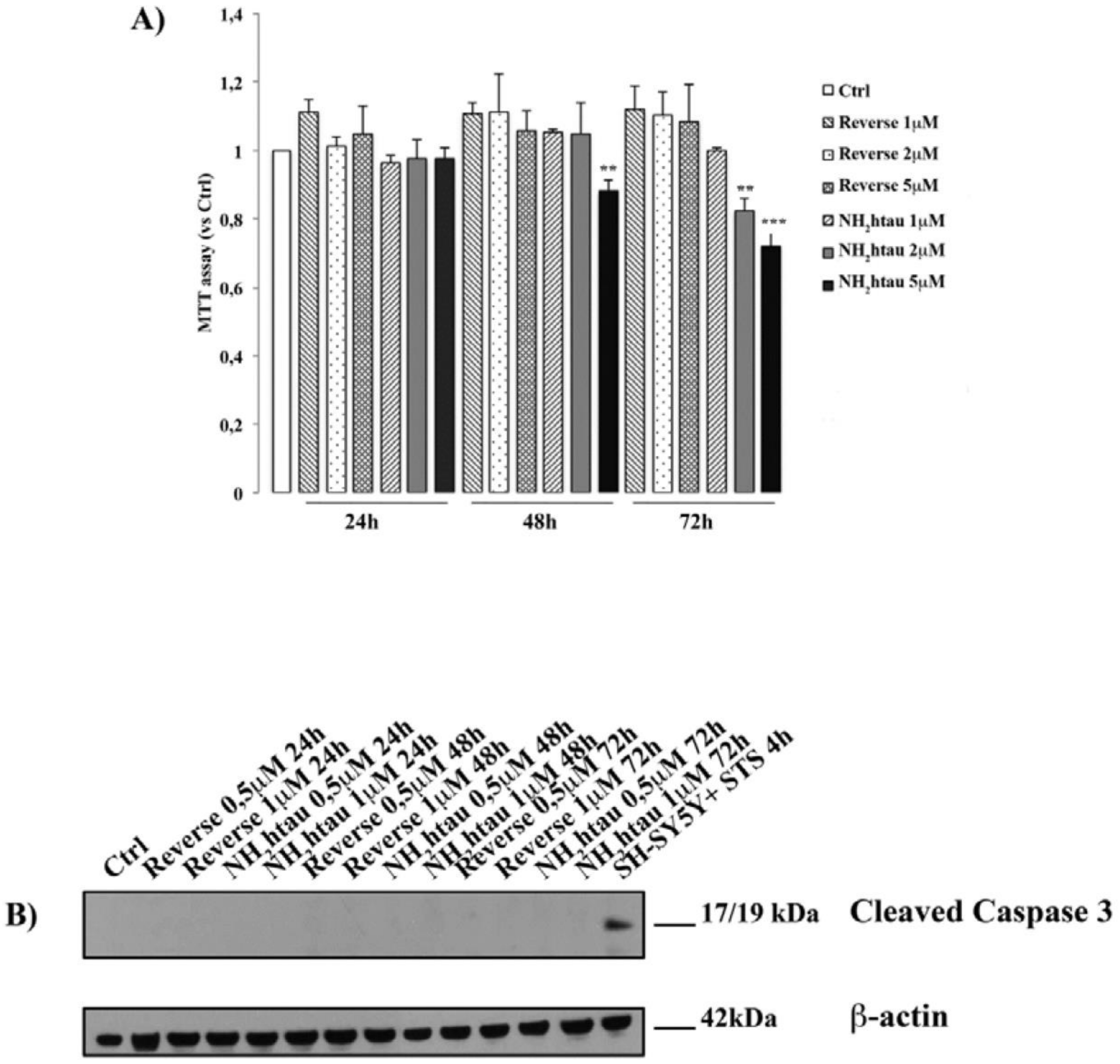

Supplementary Figure 3: Time-dependent effects of increasing concentrations of NH_2htau and its reverse peptide on cell viability of hippocampal primary neurons.

A) Mature (15 DIV) hippocampal primary neurons were exposed to different concentrations (1-2-5μM) of both NH_2htau and its reverse control sequence and cell viability were assessed by MTT assay after 24-48-72 h incubation. At 1-2μM neither NH_2htau nor reverse peptide were toxic to the cultures at any time point up to 72h. Note that only long-term treatment (72h) with higher doses (2-5μM) of extracellularly-added NH_2 26-44 leads to neuronal death. The histogram reports the neuronal survival calculated as the ratio of treated samples over untreated controls. Values are means at least of five independent experiments and statistically significant differences were calculated by unpaired two-tailed t-Student's test (**p<0,01 and *** p<0,0001 versus untreated ctrl).

B) Western blotting analysis (n=3) was carried out on equal proteins amount of total protein extract (50μg) from mature hippocampal primary neurons (DIV15) exposed for 24-48-72h to increasing concentration (0.5-1μM) of NH_2htau and its reverse sequence control. Immunoblots were probed with antibodies against the active (cleaved) form of caspase-3 (Asp175). SY5Y cells exposed to 1 μM staurosporine (STS) for 4 h and β-actin were used as positive and loading controls, respectively. Cropped representative WB are shown.

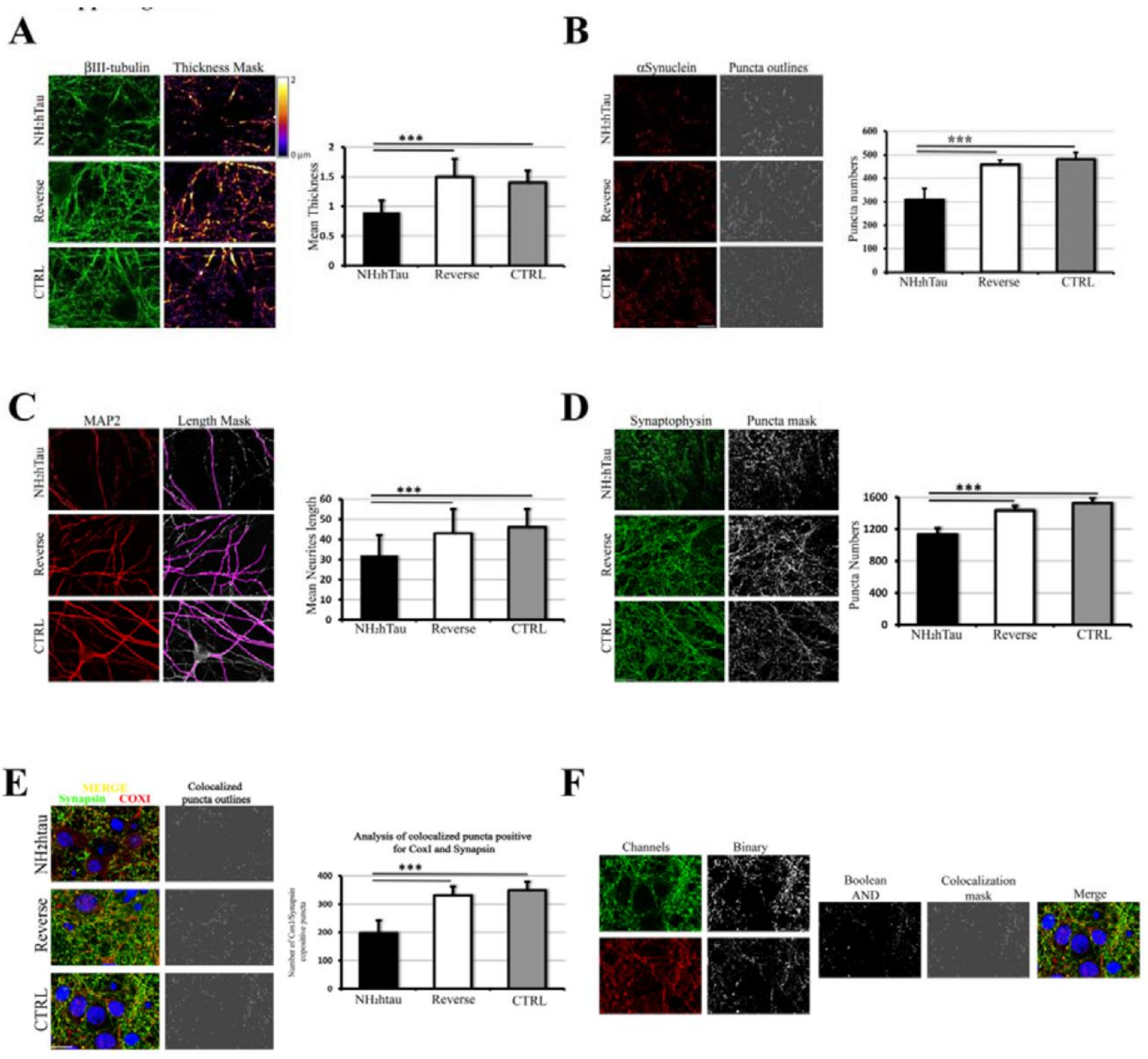

Supplementary Figure 4: Quantification of immunofluorescence data

Quantitative analysis of double immunofluorescence carried out on mature hippocampal primary neurons (DIV15) exposed for 48h to NH_2htau and its reverse control sequence (1μM).

The panels show in the first column the raw (immunofluorescence) image, in the second column the mask binary image derived from the raw image. The histograms show the quantification performed on the mask image and expressed as mean ± SD, the three groups were statistically compared by one-way repeated-measures ANOVA followed by Bonferroni post-hoc test.

A) Quantification of βIII-Tubulin immunoreactivity shows a significant decrease (about 35% vs CTRL) of the average neuritic caliber in hippocampal neurons after NH_2htau treatment (*** p<0,0001).

B) Quantification of α-synuclein-positive puncta shows a significant decrease (about 37% vs CTRL) of the average puncta number in hippocampal neurons after NH_2htau treatment (*** $p<0,0001$).

C) Quantification of MAP2 neuritic length shows a significant decrease (about 31% vs CTRL) of the average neuritic length in hippocampal neurons after NH_2htau treatment (*** $p<0,0001$).

D) Quantification of synaptophysin immunoreactive puncta shows a significant decrease (about 25% vs CTRL) of the average puncta number in hippocampal neurons after NH_2htau treatment (*** $p<0,0001$).

E) Quantification of COX-1/Synapsin double-labeled dots shows a significant decrease (about 43% vs CTRL) of the average colocalization puncta number in hippocampal cultures after NH_2htau treatment(*** $p<0,0001$).

F) The scheme shows the steps of the analytical approach used to analyze COX-I/Synapsin colocalization puncta. Scale bars: 5μm.

A)

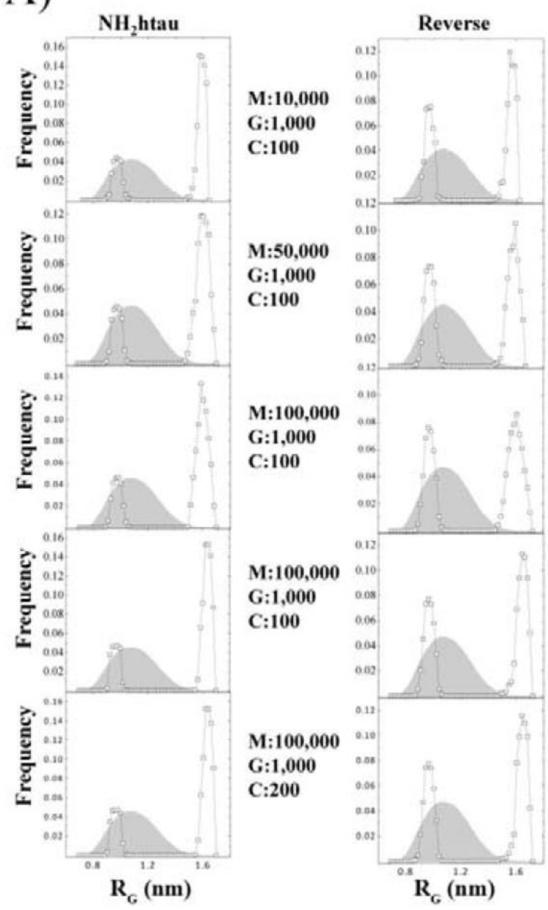

B)

	Models	Generation	Cycles	$A_{1.5}/A_1$
NH_2tau	10000	1000	100	3.00
	50000	1000	100	3.30
	100000	10000	100	3.00
	100000	10000	200	3.01
Reverse	10000	1000	100	1.48
	50000	1000	100	1.47
	100000	10000	100	1.51
	100000	10000	200	1.50

Supplementary Figure 5: Stability of the EOM fitting procedure.

A) EOM original pools (gray shaded curve) and selected pools for both NH_2tau (left panels)and its reverse sequence (right panels) are reported . The EOM fitting parameters are shown. In detail, we indicated with the "M" the total numbers of model generated by Ranch, with "G" the number of generations the genetic algorithm can use to optimize the ensemble, with "C" the number of times the genetic algorithm process was repeated.

B) EOM fitting parameters used for both peptides (columns 1-3) are shown; $A_{1.5}/A_1$ was calculated by dividing the area of the peak at high R_G by that of the peak at low R_G value.
